# A combined experimental and computational approach to unravel degradation mechanisms in electrochemical wastewater treatment†

**DOI:** 10.1039/d3ew00784g

**Published:** 2024-01-19

**Authors:** Sara Feijoo, Simona Baluchová, Mohammadreza Kamali, Josephus G. Buijnsters, Raf Dewil

**Affiliations:** a KU Leuven, Department of Chemical Engineering, Process and Environmental Technology Lab Jan Pieter de Nayerlaan 5 2860 Sint-Katelijne-Waver Belgium raf.dewil@kuleuven.be; b Delft University of Technology, Department of Precision and Microsystems Engineering Mekelweg 2 2628 CD Delft The Netherlands; c University of Oxford, Department of Engineering Science Parks Road Oxford OX1 3PJ UK

## Abstract

Electrochemical wastewater treatment is a promising technique to remove recalcitrant pollutants from wastewater. However, the complexity of elucidating the underlying degradation mechanisms hinders its optimisation not only from a techno-economic perspective, as it is desirable to maximise removal efficiencies at low energy and chemical requirements, but also in environmental terms, as the generation of toxic by-products is an ongoing challenge. In this work, we propose a novel combined experimental and computational approach to (i) estimate the contribution of radical and non-radical mechanisms as well as their synergistic effects during electrochemical oxidation and (ii) identify the optimal conditions that promote specific degradation pathways. As a case study, the distribution of the degradation mechanisms involved in the removal of benzoic acid (BA) *via* boron-doped diamond (BDD) anodes was elucidated and analysed as a function of several operating parameters, *i.e.*, the initial sulfate and nitrate content of the wastewater and the current applied. Subsequently, a multivariate optimisation study was conducted, where the influence of the electrode nature was investigated for two commercial BDD electrodes and a customised silver-decorated BDD electrode. Optimal conditions were identified for each degradation mechanism as well as for the overall BA degradation rate constant. BDD selection was found to be the most influential factor favouring any mechanism (*i.e.*, 52–85% contribution), given that properties such as its boron doping and the presence of electrodeposited silver could dramatically affect the reactions taking place. In particular, decorating the BDD surface with silver microparticles significantly enhanced BA degradation *via* sulfate radicals, whereas direct oxidation, reactive oxygen species and radical synergistic effects were promoted when using a commercial BDD material with higher boron content and on a silicon substrate. Consequently, by simplifying the identification and quantification of underlying mechanisms, our approach facilitates the elucidation of the most suitable degradation route for a given electrochemical wastewater treatment together with its optimal operating conditions.

## Abstract 2

Understanding electrochemical oxidation mechanisms through a combined experimental and computational approach.

## Graphical abstract

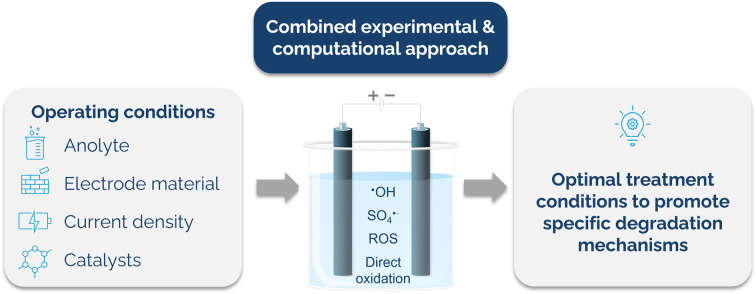

**Water impact**

Understanding the fundamentals of electrochemical oxidation is crucial to optimise the effective removal of micropollutants from wastewater while minimising chemicals and energy use and avoiding the formation of toxic by-products. Through our novel approach, applicable to both research and industrial scenarios, the identification and quantification of underlying degradation mechanisms is considerably simplified, and as a result, it sheds light on the optimal conditions to promote specific degradation pathways.

## 1 Introduction

Recent advances in high-resolution analytical techniques have shed light on the presence of numerous contaminants of emerging concern (CECs), such as pharmaceuticals, endocrine disruptors, pesticides, perfluorinated compounds, and household chemicals, at concentrations ranging from ng L^−1^ to μg L^−1^ in natural water bodies.^1–3^ These pollutants are typically recalcitrant organic compounds, and their occurrence in the environment is due to the limitations of conventional wastewater treatment to attain their complete elimination.^2–4^ In addition, it is estimated that 80% of wastewater effluents are discharged untreated,^5^ which aggravates the adverse ecotoxicological and human health effects that CECs may cause both in the short and long term.^1,6–8^

In the research trends towards developing more effective and efficient wastewater treatment, advanced oxidation processes (AOPs) stand out for their noteworthy performance in the removal of CECs from water effluents.^9^ Hydroxyl (˙OH) and sulfate radicals (SO_4_˙^−^) are the most commonly used oxidant species in AOPs and can be generated from a suitable precursor *via* electrochemical, photochemical, sonochemical or catalytic routes.^9,10^ In recent years, SO_4_˙^−^ radicals have gained more research attention than conventional ˙OH-based treatments because of several competitive advantages, including a longer half-life, higher redox potential at neutral pH, and higher selectivity towards electron-rich organic contaminants, such as aromatics and sulfur- and nitrogen-containing molecules.^9–11^ The formation of SO_4_˙^−^ radicals typically relies on the addition of a precursor such as persulfate (S_2_O_8_^2−^, PS) or peroxymonosulfate (HSO_5_^−^, PMS), while hydrogen peroxide (H_2_O_2_) and ozone (O_3_) are common sources for ˙OH.^9,11^ Nonetheless, a combination of both radicals may be attained in electrochemical wastewater treatment *via* non-active anodes such as boron-doped diamond (BDD) electrodes, where ˙OH and SO_4_˙^−^ are generated from water (eqn (1))^12–16^ and SO_4_^2−^ ions (eqn (2) and (3)),^12–18^ respectively. Given that SO_4_^2−^ is ubiquitous in wastewater effluents,^19^ sulfate radical-based electrochemical advanced oxidation processes (SR-eAOPs) offer the possibility to operate without the addition of chemical precursors, which mitigates subsequent secondary waste streams and the discharge of sulfate-rich treated waters requiring additional polishing steps.^9,16,20^

1 BDD + H_2_O → BDD(˙OH) + H^+^ + e^−^

2 BDD + SO_4_^2−^ → BDD(SO_4_˙^−^) + e^−^

3 BDD(˙OH) + SO_4_^2−^ → BDD(SO_4_˙^−^) + OH^−^

The chemistry of ˙OH and SO_4_˙^−^ radicals has been investigated in detail, and the strengths and weaknesses of each species have been reported.^11,16^ In particular, SO_4_˙^−^ is more selective and reacts with organic pollutants *via* electron transfer mechanisms, while ˙OH is non-selective and may react *via* electrophilic addition, hydrogen abstraction and electron transfer pathways.^10,11^ As a result, the treatment efficacy and the generated degradation products can differ depending on the predominant radical species as well as on their interaction with target pollutants and other wastewater constituents.^21^ Therefore, identifying and promoting the most effective radical for a given wastewater treatment application would be of great impact, not only at the technological and economic levels due to the optimal process conditions but also from a safety and environmental perspective by avoiding the generation of harmful by-products.

Due to their unstable nature, SO_4_˙^−^ radicals are highly reactive species involved in multiple reactions, such as those forming additional ˙OH radicals (eqn (4) and (5))^12,13,16,18,22^ and other oxidative species (eqn (6)–(13))^12–18,22,23^ as well as those of their indirect generation (eqn (14)–(17)),^13–17,23^ which hinders their straightforward identification and quantification. The formation of reactive species in electrochemical advanced oxidation processes (eAOPs) is also strongly dependent on the composition of the BDD electrode, that is, on factors such as boron doping level, surface termination, roughness, sp^3^/sp^2^ ratio, and substrate material, as well as the surface interactions that occur.^12,13^ In addition, the anolyte composition, current density, presence of radical scavengers and reactor design also affect the pollutant degradation efficiency.^15^

4 SO_4_˙^−^ + H_2_O → SO_4_^2−^ + ˙OH + H^+^

5 SO_4_˙^−^ + OH^−^ → SO_4_^2−^ + ˙OH

6 SO_4_˙^−^ + ˙OH → HSO_5_^−^

7
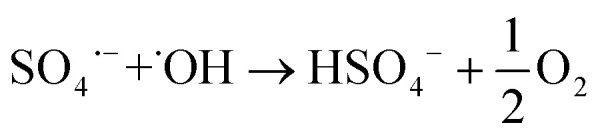

8 HSO_5_^−^ + ˙OH → SO_5_˙^−^ + H_2_O

9 HSO_5_^−^ + SO_4_˙^−^ → SO_5_˙^−^ + HSO_4_^−^

10 SO_4_^2−^ + SO_4_^2−^ → S_2_O_8_^2−^ + 2e^−^

11 SO_4_˙^−^ + SO_4_˙^−^ ↔ S_2_O_8_^2−^

12 SO_5_˙^−^ + SO_5_˙^−^ → S_2_O_8_^2−^ + O_2_

13 S_2_O_8_^2−^ + ˙OH → S_2_O_8_˙^−^ + OH^−^

14
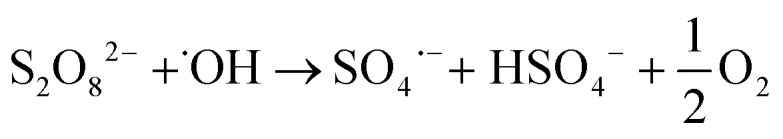

15 HSO_4_^−^ + ˙OH → SO_4_˙^−^ + H_2_O

16 SO_5_˙^−^ + SO_5_˙^−^ → SO_4_˙^−^ + SO_4_˙^−^ + O_2_

17 S_2_O_8_^2−^ + e^−^ → SO_4_˙^−^ + SO_4_^2−^

Similarly, the presence of certain transition metals behaving as catalysts may alter the reactive species formed. To this end, a literature review on metal-based catalysts for the activation of electrogenerated S_2_O_8_^2−^ ions was conducted, entailing iron-based (*e.g.*, zero-valent iron, iron oxides, and iron sulfides), copper-based (*e.g.*, zero-valent copper and copper oxides), manganese-based (*e.g.*, zero-valent manganese and manganese oxides) and other metals (*e.g.*, silver, zero-valent zinc, zero-valent aluminium, and mixed metal catalysts).^24–26^ The list of suitable metal catalysts was, however, significantly reduced when considering feasible deposition methods: only silver,^27,28^ iron^29,30^ and copper^28,31,32^ had been previously used to decorate BDD electrodes. Silver was selected for further investigation as it may react with not only S_2_O_8_^2−^ ions^25,26^ but also H_2_O_2_,^33^ both of which can be electrogenerated in a sulfate-based water medium,^12,13,34,35^ and lead to the formation of additional radicals‡‡In addition, silver was selected due to the negative environmental effects of copper^38^ and the unsuccessful deposition attained with iron (data not shown). (eqn (18)–(20)).^16,26,33^ The activation of both species may avoid their interaction (eqn (21)),^13,36^ scavenging reactions forming weaker oxidants such as hydroperoxyl radicals (HO_2_˙) (eqn (22)–(25)),^22,37^ and the formation of a persulfate-based transition state structure (eqn (26)) that is argued to further decompose into SO_4_^2−^ instead of SO_4_˙^−^.^13,18,22^ Therefore, from a fundamental chemistry point of view, it is interesting to explore the effects of decorating the BDD surface with a metal catalyst due to the potential variations in underlying degradation mechanisms, even if lowering the overall performance and cost-effectiveness of the BDD material.

18 Ag^+^ + S_2_O_8_^2−^ → Ag^2+^ + SO_4_˙^−^ + SO_4_^2−^

19 Ag^+^ + S_2_O_8_^2−^ → [Ag^II^(SO_4_˙)]^+^ + SO_4_^2−^

20 Ag + H_2_O_2_ + H^+^ → Ag^+^ + ˙OH + H_2_O

21 H_2_O_2_ + S_2_O_8_^2−^ → 2SO_4_^2−^ + 2H^+^ + O_2_

22 SO_4_˙^−^ + H_2_O_2_ → SO_4_^2−^ + H^+^ + HO_2_˙

23 SO_4_˙^−^ + HO_2_˙ → SO_4_^2−^ + H^+^ + O_2_

24 ˙OH + H_2_O_2_ → H_2_O + HO_2_˙

25 ˙OH + HO_2_˙ → O_2_ + H_2_O

26 SO_4_˙^−^ + S_2_O_8_^2−^ → SO_4_^2−^ + S_2_O_8_˙^−^

To unravel the reactive species participating in pollutant degradation processes, several analytical techniques, such as electron paramagnetic resonance (EPR) and reactions with chemical probes, and computational tools, such as quantum chemical calculations and machine learning models, have been explored.^11,15,39^ Given the complexity of applying these advanced methods, the development of standard approaches that combine experimental and computational procedures to estimate the contributions of the different reactive species would make the understanding of eAOP fundamentals more accessible and ready to be applied in real case scenarios. Several studies have proposed kinetic models to explain the formation of SO_4_˙^−^ and ˙OH radicals in photocatalytic wastewater treatment.^23,40–42^ However, the electrochemical oxidation field still lacks such a model, where not only the degradation mechanisms *via* SO_4_˙^−^ and ˙OH radicals are evaluated, but also other reactive species and their potential synergistic effects are included.^15^

Consequently, this work focused on developing a comprehensive method that (i) estimates the distribution of multiple radical and non-radical degradation mechanisms as well as their synergistic effects during the SR-eAOP treatment and (ii) sheds light on the optimal conditions to promote specific degradation pathways. As a novelty, our combined experimental and computational approach circumvents the need for advanced analytical and simulation techniques, making the understanding and optimisation of SR-eAOPs available to a wider research and industry audience. Our approach was first applied to the degradation of a chemical probe (*i.e.*, benzoic acid (BA)) and analysed as a function of several operating parameters, namely, the initial sulfate (SO_4_^2−^) and nitrate (NO_3_^−^) content of the wastewater and the current applied. Subsequently, a multivariate optimisation study was conducted, where in addition the influence of the electrode nature was investigated for two commercial BDD electrodes and a customised silver-decorated BDD electrode. As a result, the optimal operating conditions for each degradation mechanism as well as for the overall BA degradation rate constant were identified.

## 2 Materials & methods

### 2.1 Chemicals

High-purity grade benzoic acid (C_6_H_5_COOH, BA, 99.6%) was purchased from Acros Organics (Belgium). Nitrobenzene (C_6_H_5_NO_2_, NB, 99%) and silver nitrate (AgNO_3_, 99%) were obtained from Sigma-Aldrich (Germany). Acetonitrile (CH_3_CN, 99.9%), ammonium molybdate tetrahydrate ((NH_4_)_6_Mo_7_O_24_·4H_2_O, 99%), *tert*-butanol ((CH_3_)_3_COH, 99.5%), methanol (CH_3_OH, UHPLC grade), nitric acid (HNO_3_, 70%), potassium hydroxide (KOH, pure), potassium persulfate (K_2_S_2_O_8_, reagent grade), sodium bicarbonate (NaHCO_3_, 99.5%), sodium nitrate (NaNO_3_, 99%) and sodium sulfate (Na_2_SO_4_, 99%) were acquired from Acros Organics (Belgium). Acetic acid (CH_3_COOH, ≥99.9%), orthophosphoric acid (H_3_PO_4_, 85%), potassium iodide (KI, ≥99.5%) and sodium acetate trihydrate (NaCH_3_COO·3H_2_O, ≥99%) were purchased from VWR Chemicals (Belgium). Potassium ferrocyanide trihydrate (K_4_Fe(CN)_6_·3H_2_O, ≥98%) was obtained from Alfa Aesar (Germany). Working solutions were prepared with Milli-Q water purified using a Milli-Q®-Reference system (18 MΩ cm) from Merck (Germany).

### 2.2 Electrode preparation

Two commercial working electrodes were used in this study: (i) a polycrystalline BDD coating (12 μm thickness) on a 40 × 80 × 2 mm^3^ monocrystalline niobium plate (Magneto Special Anodes, The Netherlands), further referred to as “Nb/BDD”, and (ii) a polycrystalline BDD coating (5–10 μm thickness, ∼5000 ppm boron doping) on a 40 × 80 × 3 mm^3^ monocrystalline silicon plate (Redox.me, Sweden), further referred to as “Si/BDD”. A duplicate of Nb/BDD was used for in-house surface decoration with silver particles, further referred to as “Nb/BDD-Ag”. The decoration of Nb/BDD-Ag was carried out through electrodeposition based on the method reported by Jiang *et al.* (2015).^27^ A fixed potential of −0.1 V *vs.* Ag/AgCl was applied for 300 s in aqueous electrolyte containing 10 mM silver nitrate (AgNO_3_) and 0.2 M acetic acid/sodium acetate buffer. Given the satisfactory deposition results and the lower tendency observed towards forming silver particle agglomerates, the use of cetyltrimethylammonium bromide (CTAB, C_19_H_42_BrN) as a surfactant was avoided in contrast to the original method. After each electrodeposition and between degradation experiments, the electrode was rinsed with Milli-Q water. The Nb/BDD-Ag electrode was reused for each set of experimental conditions up to a maximum of 2 h of total operation to avoid jeopardising the degradation efficiency. An exception was applied for the experiments where methanol was present, since the degradation efficiency *via* the modified electrode surface dramatically changed after 1 h. The Nb/BDD-Ag electrode was submerged in nitric acid (HNO_3_) for 30 min to ensure complete removal of silver deposits before each new electrodeposition.

### 2.3 Electrode characterisation

The surface morphology of the electrodes was visualised with a JEOL JSM-6010LA scanning electron microscope (SEM) (JEOL, USA) operated in secondary electron imaging mode at 20 keV. The elemental composition of the silver-decorated BDD electrode was analysed with a high-resolution JEOL JSM-6500F SEM coupled with a JEOL energy dispersive X-ray spectrometer (EDS) (JEOL, USA). The corresponding microscopic image analysis was conducted with ImageJ software version 1.53 t.^43^ Surface roughness was estimated through Gwyddion software^44^ from the measurements performed with a Nanosurf Nanite B atomic force microscope (AFM) (Nanosurf, Switzerland) operated in a contact mode over a scanned area of 10 × 10 μm^2^. Raman spectra were measured with a Horiba LabRAM HR device (Horiba Scientific, Japan) equipped with an argon-ion laser operating at 514 nm wavelength.

The ferrocyanide redox couple (eqn (27)) was used to determine the active surface area of each electrode by means of cyclic voltammetry (CV) as reported by Zhu and Zhao (2019),^45^ which was performed by potential sweeping from −0.2 V to +0.7 V (*vs.* Ag/AgCl reference electrode) at a scan rate of 0.1 V s^−1^ for 4 cycles. Based on the peak current (*I*_p_, A) observed in the cyclic voltammogram, the effective anode area (*A*_e_, cm^2^) was calculated from the Randles–Sevcik equation (eqn (28)), where *n* is the number of electrons transferred in the redox reaction (*i.e.*, 1), *D* is the diffusion coefficient (*i.e.*, 6 × 10^−6^ cm^2^ s^−1^ for ferrocyanide^45^), *C* is the concentration of the reaction species (*i.e.*, 10^−6^ mol cm^−3^ ferrocyanide), and *v* is the applied scan rate (*i.e.*, 0.1 V s^−1^). These CV experiments also enabled the calculation of the mass transfer coefficient (*k*_m_, m s^−1^) according to eqn (29), where *F* corresponds to the Faraday constant (*i.e.*, 96 485 C mol^−1^).^46^

27 [Fe(CN)_6_]^4−^ → [Fe(CN)_6_]^3−^ + e^−^

28
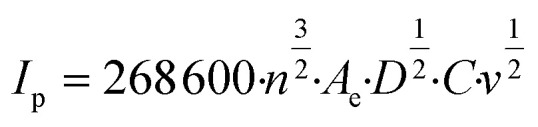
29
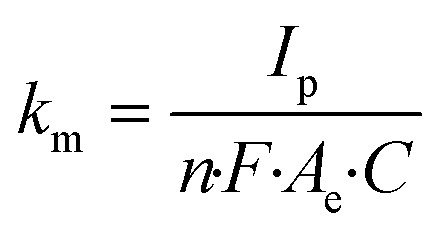


For each degradation scenario, the limiting current density (*j*_lim_, A m^−2^) for the mineralisation of organics was estimated through eqn (30), where *k*_m_ is obtained as shown in eqn (29), and COD(*t*) is the chemical oxygen demand (mol O_2_ m^−3^) at the start of the experiment.^47^

30 *j*_lim_(*t*) = 4·*F*·*k*_m_·COD(*t*)

### 2.4 Experimental setup

Degradation experiments were performed in a 1 L undivided laboratory-scale electrochemical cell (Redox.me, Sweden), where the anodes described in Section 3.2 were used to carry out the oxidation reactions. The counter electrode was a commercial stainless steel 317 L electrode with dimensions of 40 × 80 × 2 mm^3^, and the reference electrode selected was Ag/AgCl (+0.210 V *vs.* SHE), placed in the vicinity of the working electrode (Redox.me, Sweden). The interelectrode distance was fixed at 40 mm. Chronopotentiometry experiments were conducted with a potentiostat/galvanostat AUT302N.S PGSTAT302N acquired from Metrohm (Belgium), including a pH/temperature module (pX1000.S). At given time intervals, 1 mL samples were collected, filtered with a high-grade syringe filter (CHROMAFIL® Xtra PET, 25 mm diameter, 0.2 μm pore size, Macherey-Nagel, Germany) and subsequently quenched with 500 μL UHPLC-grade methanol to prevent further degradation reactions. Experiments were conducted in batch mode, at room temperature, and with constant magnetic stirring at 600 rpm. To ensure the reproducibility and reliability of the results, target experiments were performed at least in triplicate, and complementary tests were conducted at least in duplicate.

### 2.5 Kinetic model & design of experiments

Benzoic acid (BA) is a known chemical probe for both ˙OH and SO_4_˙^−^ radicals, with second order rate constants of 5.9 × 10^9^ M^−1^ s^−1^ and 1.2 × 10^9^ M^−1^ s^−1^, respectively.^20,40^ Likewise, nitrobenzene (NB) is a chemical probe assumed to react only with ˙OH at a rate of 3.9 × 10^9^ M^−1^ s^−1^ since its reaction with SO_4_˙^−^ is considerably slower (<10^6^ M^−1^ s^−1^).^20,40^ By investigating the competition kinetics of both compounds, researchers have previously proposed a model to estimate the steady-state concentrations of ˙OH and SO_4_˙^−^ radicals (denoted as [˙OH]_ss_ and [SO_4_˙^−^]_ss_) according to eqn (31) and (32), where *k*_obs,BA_ and *k*_obs,NB_ correspond to the pseudo-first order kinetics observed during the degradation of BA and NB, respectively.^40,48^

31 *k*_obs,BA_ = 5.9 × 10^9^·[˙OH]_ss_ + 1.2 × 10^9^·[SO_4_˙^−^]_ss_

32 *k*_obs,NB_ = 3.9 × 10^9^·[˙OH]_ss_

When simultaneously degrading BA and NB in the presence of SO_4_˙^−^ ions in the electrochemical cell of this study, it was observed that NB was degraded at a faster rate than BA (Fig. A.1, ESI†). Given that the above kinetic model would expect *k*_obs,BA_ to be greater than *k*_obs,NB_, the discrepancies with the experimental observations infer that there may be other species involved as well as possible scavenging reactions. Consequently, an extended kinetic model comprising radical and non-radical degradation mechanisms was proposed to quantify their contributions (denoted by ∥ in eqn (33)) to the observed BA pseudo-first order kinetic constant in a sulfate- and nitrate-based medium.

33 *k*_obs,BA_ = |SO_4_˙^−^| + |*A*_ox_| + |S_2_O_8_^2−^| + |H_2_O_2_| + |ROS| + |*λ*_nat_| + |*λ*_SO_4^2−^__| + |*λ*_NO_3^−^__| + |*S*_˙OH,SO_4˙^−^__| + |*S*_˙OH,NO_3^−^__| + |*S*_SO_4˙^−^,NO_3_^−^__|

In our proposed model, |SO_4_˙^−^| refers to the degradation *via* SO_4_˙^−^ radicals that are generated from both initial SO_4_^2−^ ions (eqn (2)) and electrogenerated S_2_O_8_^2−^ ions (eqn (11) and (17)), while the synergy term |*S*_˙OH,SO_4_˙^−^_| comprises both the degradation *via* ˙OH radicals formed at the BDD anode (eqn (1)) and the subsequent reactions involving both SO_4_˙^−^ and ˙OH radicals present in solution (eqn (3)–(9) and (12)–(16)). Non-radical mechanisms include the direct oxidation of the pollutant on the BDD surface (|*A*_ox_|) and degradation *via* electrogenerated species, such as persulfate (|S_2_O_8_^2−^|), hydrogen peroxide (|H_2_O_2_|), and reactive oxygen species (|ROS|), the latter comprising primarily singlet oxygen (^1^O_2_) since HO_2_˙ radicals are not formed at high potentials due to the oxygen evolution reaction (OER).^49^ The influence of adding NO_3_^−^ ions to the electrolyte is represented by |*S*_˙OH,NO_3_^−^_| and |*S*_SO_4_˙^−^,NO_3_^−^_|, depending on whether interactions with ˙OH (eqn (34)) or SO_4_˙^−^ (eqn (35)) occur,^13,18,39^ respectively. As a result, nitrate radicals (NO_3_˙) may be generated in the system, presenting a lower standard redox potential (up to +2.49 V^50^) than ˙OH (up to +2.85 V^51^) and SO_4_˙^−^ (up to +3.10 V^52^). Photodegradation by natural light (|*λ*_nat_|) and by photoactivated sulfate (|*λ*_SO_4_^2−^_|) and nitrate (|*λ*_NO_3_^−^_|) ions is also considered. When elucidating each contributor in the model, a negative value represents that scavenging reactions are predominant, whereas a positive value reflects an enhancing effect on pollutant oxidation.

34 ˙OH + NO_3_^−^ → NO_3_˙ + OH^−^

35 SO_4_˙^−^ + NO_3_^−^ → NO_3_˙ + SO_4_^2−^

To implement our kinetic model, a tailored experimental approach was developed, where a specific set of experiments enabled the elucidation of the different contributions in a cascade manner (Table 1). The selection of methanol and *tert*-butanol as radical scavengers in experiments no. 8 and 11 was made based on their reported quenching properties.^39^ For species such as H_2_O_2_ and S_2_O_8_^2−^, additional experiments (*i.e.*, no. 7, 9, and 10) were conducted to estimate the potential inhibition effect of both these scavengers. Under these quenched scenarios, the scavenger : pollutant molar ratio was set to 1000 : 1.^49^ In addition, sodium acetate was added in experiment no. 8 to increase the conductivity of the medium and enable the quantification of the direct oxidation and ROS contributions. It was selected as background electrolyte for several reasons: (i) its reaction rate constants with ˙OH and SO_4_˙^−^ radicals in comparison to those of BA are 2 and 3 orders of magnitude lower, respectively,^20,40,53,54^ (ii) experimental assays showed that BA was not naturally degraded in an acetate-based solution (data not shown), (iii) electrolytes containing halides such as chloride ions (Cl^−^) could lead to the formation of additional oxidants (*e.g.*, Cl˙, Cl_2_, Cl_2_O, ClO_2_, and HOCl) as well as toxic by-products (*e.g.*, chlorate (ClO_3_^−^) and perchlorate (ClO_4_^−^)),^55–58^ and (iv) other species such as carbonate (CO_3_^2−^), bicarbonate (HCO_3_^−^) and phosphate (PO_4_^3−^) ions are known scavengers that could interfere with the degradation.^39^ Since methanol was also added in experiment no. 8 to already scavenge ˙OH radicals, it can be argued that any plausible ˙OH scavenging effect by sodium acetate did not affect the implementation of the kinetic model. In a real wastewater scenario, the use of acetate could be omitted if the influent wastewater presented a sufficient conductivity level. As a result, it would be also possible to quantify the contribution of ˙OH radicals alone.

**Table 1:** Combined experimental and computational approach to unravel the distribution of degradation mechanisms in a sulfate- and nitrate-based medium

Exp.	Operating conditions	Model implementation	Calculated parameter
1	BA photodegradation in pure water (no current applied)	*k*_BA,1_ = ∣*λ*_nat_∣	|*λ*_nat_|
2	BA photodegradation in SO_4_^2−^ medium (no current applied)	*k*_BA,2_ = |*λ*_nat_| + |*λ*_SO_4_^2−^_|	|*λ*_SO_4_^2−^_|
3	BA photodegradation in SO_4_^2−^ and NO_3_^−^ medium (no current applied)	*k*_BA,3_ = |*λ*_nat_| + |*λ*_SO_4_^2−^_| + |*λ*_NO_3_^−^_|	|*λ*_NO_3_^−^_|
4	Electrogeneration of H_2_O_2_ and S_2_O_8_^2−^ in SO_4_^2−^ and NO_3_^−^ medium without BA (current applied)	Not applicable	[H_2_O_2_], [S_2_O_8_^2−^]
5	BA degradation in S_2_O_8_^2−^ medium with initial concentration as quantified in exp. 4 (no current applied)	*k*_BA,5_ = |S_2_O_8_^2−^| + |*λ*_nat_|	|S_2_O_8_^2−^|
6	BA degradation in H_2_O_2_ medium with initial concentration as quantified in exp. 4 (no current applied)	*k*_BA,6_ = |H_2_O_2_| + |*λ*_nat_|	|H_2_O_2_|
7	Same as exp. 6 with added methanol to calculate the scavenging effect (%) to H_2_O_2_	*k*_BA,7_ = |H_2_O_2_|·(1 − *S*_M/H_2_O_2__) + |*λ*_nat_|	*S*_M/H_2_O_2__
8	BA degradation in pure water with methanol as scavenger for ˙OH radicals (current applied). Sodium acetate was added to increase the conductivity	*k*_BA,8_ = |*A*_ox_| + |H_2_O_2_|·(1 − *S*_M/H_2_O_2__) + |ROS| + |*λ*_nat_|	|*A*_ox_| + |ROS|
9	Same as exp. 5 with added tert-butanol to calculate the scavenging effect (%) to S_2_O_8_^2−^	*k*_BA,9_ = |S_2_O_8_^2−^|·(1 − *S*_T/S_2_O_8_^2−^_) + |*λ*_nat_|	*S*_T/S_2_O_8_^2−^_
10	Same as exp. 6 with added *tert*-butanol to calculate the scavenging effect (%) to H_2_O_2_	*k*_BA,10_ = |H_2_O_2_|·(1 − *S*_T/H_2_O_2__) + |*λ*_nat_|	*S*_T/H_2_O_2__
11	BA degradation in SO_4_^2−^ medium with tert-butanol as scavenger for ˙OH radicals (current applied)	*k*_BA,11_ = |SO_4_˙^−^| + |*A*_ox_| + |S_2_O_8_^2−^|·(1 − *S*_T/S_2_O_8_^2−^_) + |H_2_O_2_|·(1 − *S*_T/H_2_O_2__) + |ROS| + |*λ*_nat_| + |*λ*_SO_4_^2−^_|	|SO_4_˙^−^|
12	BA degradation in SO_4_^2−^ medium (current applied)	*k*_BA,12_ = |SO_4_˙^−^| + |*A*_ox_| + |S_2_O_8_^2−^| + |H_2_O_2_| + |ROS| + |*λ*_nat_| + |*λ*_SO_4_^2−^_| + |*S*_˙OH, SO_4_˙^−^_|	|*S*_˙OH, SO_4_˙^−^_|
13	BA degradation in NO_3_^−^ medium (current applied)	*k*_BA,13_ = |*A*_ox_| + |H_2_O_2_| + |ROS| + |*λ*_nat_| + |*λ*_NO_3_^−^_| + |*S*_˙OH,NO_3_^−^_|	|*S*_˙OH, NO_3_^−^_|
Target	BA degradation in SO_4_^2−^ and NO_3_^−^ medium (current applied)	*k*_obs,BA_ = |SO_4_˙^−^| + |*A*_ox_| + |S_2_O_8_^2−^| + |H_2_O_2_| + |ROS| + |*λ*_nat_| + |*λ*_SO_4_^2−^_| + |*λ*_NO_3_^−^_| + |*S*_˙OH,SO_4_˙^−^_| + |*S*_˙OH,NO_3_^−^_| + |*S*_SO_4_˙^−^,NO_3_^−^_|	|*S*_SO_4_˙^−^, NO_3_^−^_|

The proposed kinetic model was first implemented to elucidate the distribution of the different degradation mechanisms involved in the removal of BA with commercial Nb/BDD. To this end, 4 scenarios with different SO_4_^2−^ and NO_3_^−^ concentrations as well as currents applied were selected (Table 2). Since no significant degradation was observed due to electrogenerated S_2_O_8_^2−^ and H_2_O_2_ (experiments no. 5 and 6) as well as due to direct and sulfate-driven photodegradation mechanisms (experiments no. 1 and 2), a simplified version of the model was implemented (Table A.1, ESI†).

**Table 2:** Scenarios investigated for the degradation of BA with the commercial Nb/BDD electrode

Scenario	Electrode	BA (μM)	Na_2_SO_4_ (mM)	NaNO_3_ (mM)	Current (mA)
A	Nb/BDD	10	10	10	90
B	Nb/BDD	10	50	10	90
C	Nb/BDD	10	50	10	180
D	Nb/BDD	10	10	50	90

Second, the Taguchi method for optimisation was applied in combination with the kinetic model to investigate the influence of multiple electrode materials and operating conditions on the degradation mechanisms. The Taguchi method is based on standard orthogonal arrays to test the most relevant combinations of process factors (*i.e.*, parameters) at distinct values (*i.e.*, levels) to identify their contribution to the optimisation target.^59^ In this study, 3 parameters (*i.e.*, electrode material, initial sulfate concentration, and applied current) were selected, of which the electrode material was defined for 3 levels, and the rest were set at 2 different levels. Consequently, a variation of the Taguchi L8b statistical design was selected (Table 3) and applied to a simplified sulfate-based kinetic model (Table A.2, ESI†).

**Table 3:** Scenarios investigated according to the Taguchi design

Taguchi test	Electrode	BA (μM)	Na_2_SO_4_ (mM)	Current (mA)
1	Nb/BDD	10	10	90
2	Nb/BDD	10	50	180
3	Si/BDD	10	50	90
4	Si/BDD	10	10	180
5	Nb/BDD-Ag	10	50	90
6	Nb/BDD-Ag	10	10	180

### 2.6 Chemical analysis

The concentrations of BA and NB were measured simultaneously with an ultra-high performance liquid chromatography instrument (1260 Infinity II UHPLC, Agilent Technologies, Germany). The detection method was based on the high performance liquid chromatography (HPLC) method developed by Schmid *et al.* (2008),^60^ whose parameters were translated to an UHPLC equivalent.^61^ After validating the suitability of the method parameters, chromatographic separations were carried out by injecting 20 μL samples through the Agilent InfinityLab Poroshell 120 EC-C18 column (2.1 × 50 mm, 1.9 μm), operated at a flow rate of 0.5 mL min^−1^ and at 30 °C. The gradient elution program with mobile phases (A) Milli-Q water with 10 mM phosphoric acid (pH 2.3) and (B) pure acetonitrile started at 27% of B for 1 min, followed by an increase to 40% in 1.9 min, which was then maintained for 0.5 min. Afterwards, it returned to the initial conditions in 0.1 min, which were kept constant for 1.5 min. UV detection was performed at wavelengths of 228 and 266 nm for BA and NB, respectively. In the degradation experiments with only BA, the isocratic method by Sun *et al.* (2022)^40^ was used as a reference, where a mixture of 75% mobile phase (A) Milli-Q water with 10 mM phosphoric acid (pH 2.3) and 25% (B) methanol was run at 0.5 mL min^−1^ and 30 °C for 4 min, and BA detection was carried out at 230 nm.

The formation of persulfate was monitored based on the spectrophotometric method developed by Liang *et al.* (2008).^62^ Without quenching, 1 mL of the reaction sample was added to 1 mL of a solution containing 5 g L^−1^ sodium bicarbonate (NaHCO_3_) and 100 g L^−1^ potassium iodide (KI). The reaction mixture was homogenised in a closed transparent tube. After 15 min, its absorbance was measured with a UV-2600 UV-VIS spectrophotometer (Shimadzu, Japan) at a wavelength of 350 nm. The formation of hydrogen peroxide was quantified *via* the Ghormley triiodide method^63^ and with the addition of ammonium molybdate as a catalyst.^64^ To this end, 0.2 mL of reaction sample was added to 0.2 mL of 1 M KI and 2 mL of a mixture containing 0.1 M acetic acid/sodium acetate and 0.3 mM ammonium molybdate. After 1 min, the absorbance was analysed in the UV-VIS spectrophotometer at a wavelength of 350 nm. For both S_2_O_8_^2−^ and H_2_O_2_ quantification tests, experiments were performed without the presence of the target pollutant. Chemical oxygen demand (COD) was measured with the commercial Hach reagent kit LCK1414 and the DR3900 laboratory spectrophotometer (Hach, Germany).

### 2.7 Data analysis

Degradation kinetics were modelled under pseudo-first order and fitted by non-linear regressions.^49^ The optimisation of the different operating parameters to the distribution of degradation mechanisms and the predicted optimal results were investigated through the Taguchi method in combination with analysis of variance (ANOVA), as reported previously.^49^ The optimisation target was set to maximise the contribution of specific degradation mechanisms as well as the overall BA degradation rate constant. Therefore, the signal-to-noise ratio (S/N) was calculated under the “higher-the-better” definition (eqn (36)), where *y* and *n* are the experimental observations and their number, respectively.^65^ Calculations were performed with RStudio® software version 1.4.1103.^66^36
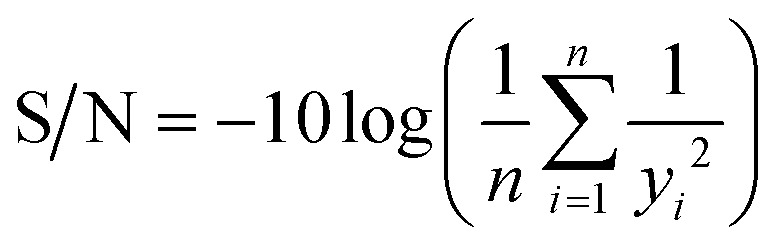


## 3 Results

### 3.1 Characterisation: commercial BDD electrodes

**Fig. 1:**
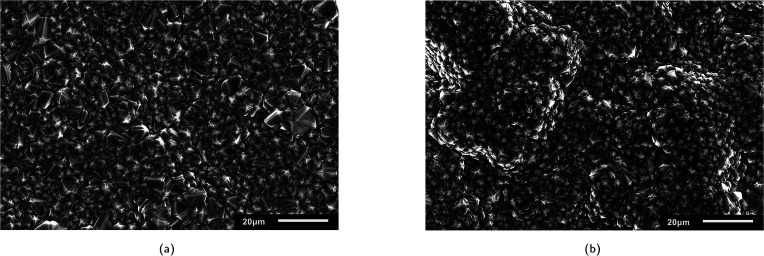
SEM images of commercial (a) Nb/BDD and (b) Si/BDD electrodes.

**Fig. 2:**
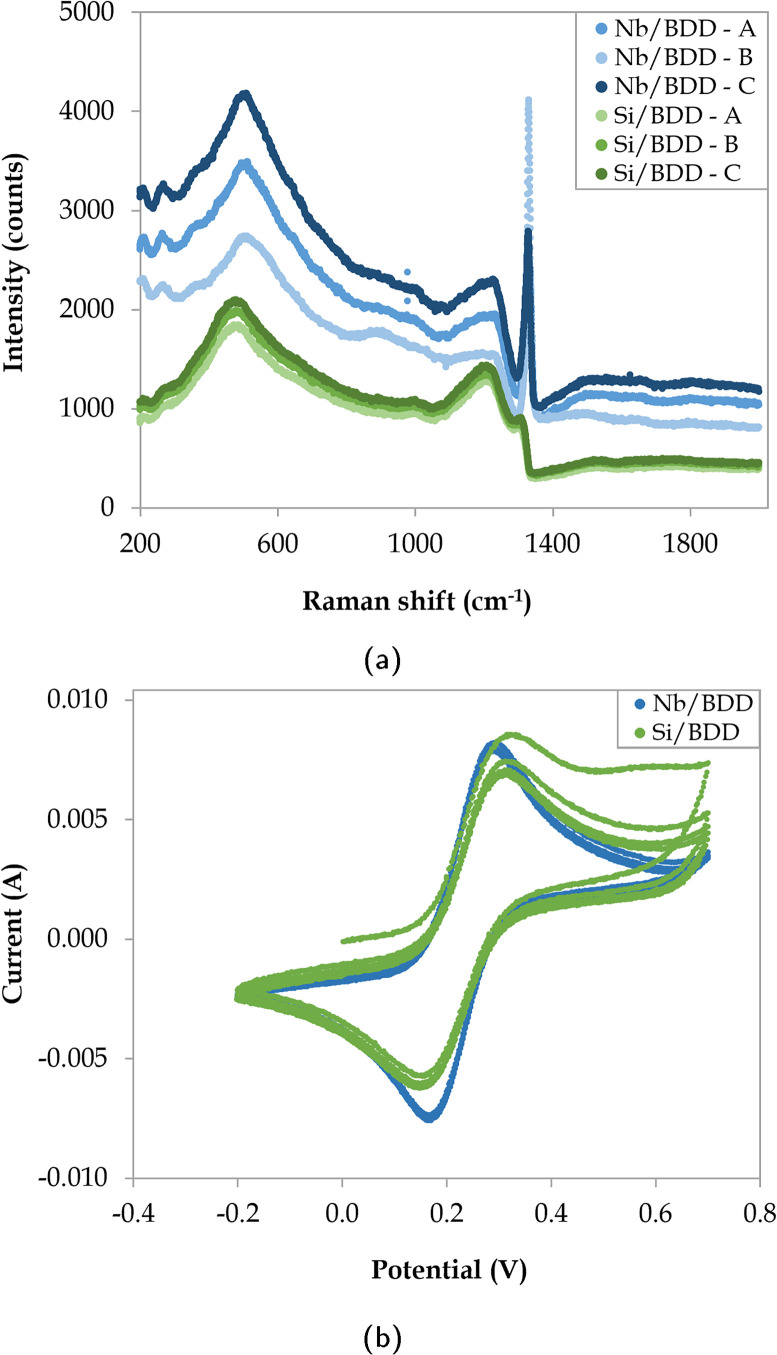
(a) Raman spectra at 3 different locations (A, B, and C) and (b) CV experiments (4 cycles) for Nb/BDD and Si/BDD electrodes.

The surface morphology of the two commercial BDD electrodes was investigated through SEM analysis (Fig. 1), where both electrodes exhibited randomly oriented crystallites with predominant {111} triangular facets and doubled edges, which likely resulted from crystal twinning. Notably, Nb/BDD exhibited a flatter arrangement of crystals with a heterogeneous size distribution (Fig. 1a), while Si/BDD presented overall smaller grain sizes constituting a cauliflower-type surface morphology^67^ (Fig. 1b). The roughness of each electrode was calculated based on AFM measurements (Fig. B.1, ESI†) and averaged at several locations, where 596 ± 120 nm and 512 ± 23 nm corresponded to the root mean square (RMS) roughness for Nb/BDD and Si/BDD, respectively. To resolve the composition of both BDD electrodes, Raman spectroscopic analysis was conducted. As depicted in Fig. 2a, the Raman spectra recorded from Nb/BDD presented a sharp diamond peak located at 1332 cm^−1^, whereas the Si/BDD electrode displayed typical Raman spectra of a heavily boron-doped diamond film, with strong distortion of the diamond one-phonon line into two separate branches located at 1200 and 1300 cm^−1^. Both types of electrodes showed another strong feature indicating the presence of BDD, *i.e.*, the intense broad band at approximately 480 cm^−1^, attributable to a combination of electronic Raman scattering and a Fano-shaped band.^68,69^ In relative terms, the Si/BDD electrode exhibited one order of magnitude higher boron content (*i.e.*, 2.0 × 10^21^ atoms per cm^3^) compared to the doping assessed for Nb/BDD (*i.e.*, 4.2 × 10^20^ atoms per cm^3^), as calculated from a fitting tool.^68,70^ The intensity of the G-band at *ca.* 1580 cm^−1^ (ref. 71) was rather low for both electrodes, thus indicating only a minor content of sp^2^ carbon residing at their grain boundaries. Finally, CV experiments (Fig. 2b) enabled the quantification of their active surface area and mass transfer coefficients (Table B.1, ESI†). The Nb/BDD electrode presented a higher active surface area (*i.e.*, 57.1 cm^2^) than the Si/BDD electrode (*i.e.*, 46.6 cm^2^), presumably due to its higher roughness.

### 3.2 Characterisation: silver-decorated BDD electrode

**Fig. 3:**
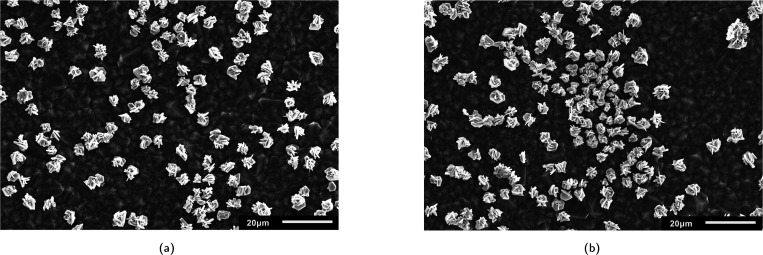
SEM images of electrodeposited Nb/BDD-Ag with (a) homogeneous and (b) heterogeneous growth patterns.

After the electrodeposition of silver, visible micro-flower structures were observed on the BDD surface *via* SEM analysis and confirmed by EDS measurements (Fig. B.2, ESI†). The distribution of the silver deposits was observed to be not entirely homogeneous, as local differences in electrode composition, operating temperature and/or magnetic stirring could have resulted in the different growth patterns. As shown in Fig. 3, several locations exhibited a homogeneous coverage of particles in terms of size, shape, and scattering (Fig. 3a), whereas other areas presented a heterogeneous distribution due to particle agglomeration (Fig. 3b). Therefore, particle size distributions and silver coverage densities were calculated over at least 12 different locations in the 5–50 μm scale range per sampling time for a more representative characterisation. AFM measurements were not conducted on this electrode, as its increased roughness triggered the immediate breakage of the cantilever.

**Fig. 4:**
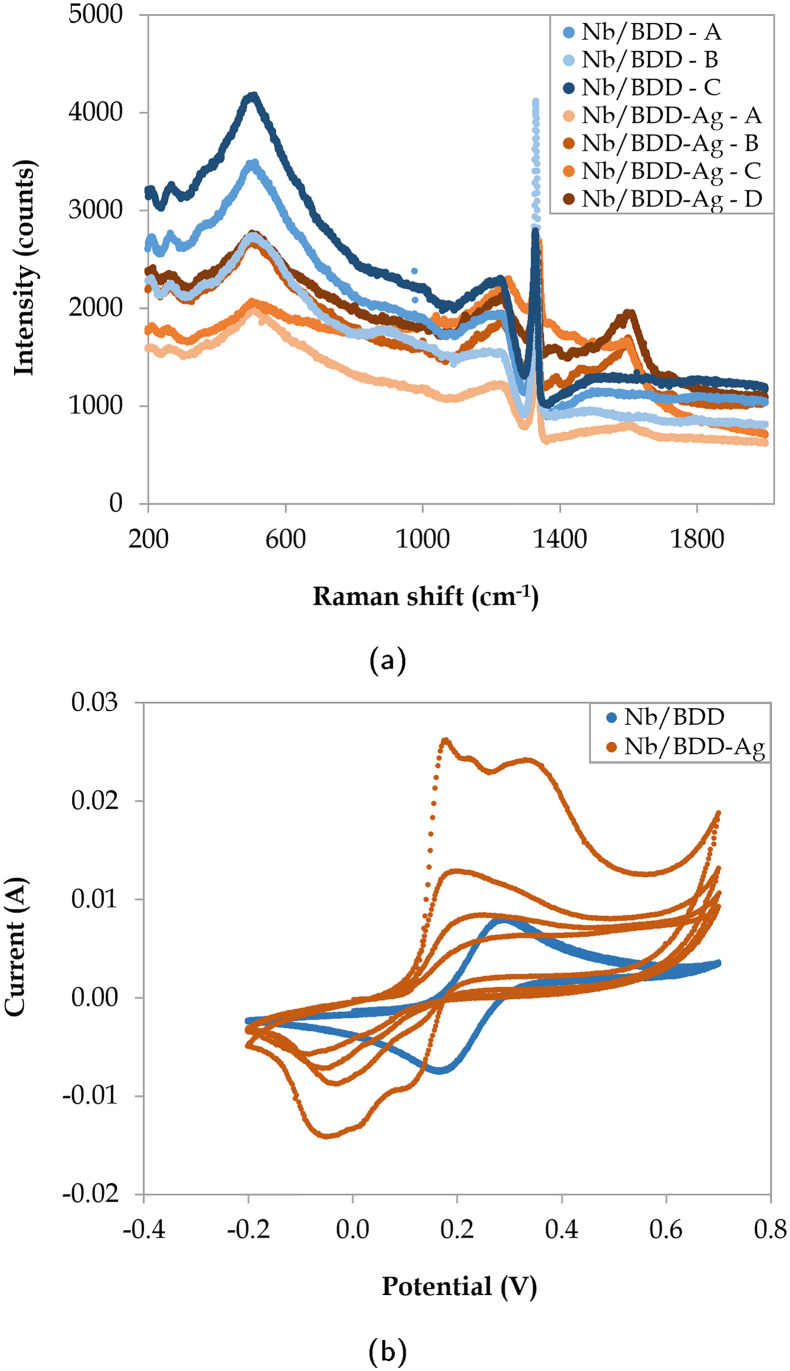
(a) Raman spectra at 4 different locations (A, B, C, and D) and (b) CV experiments (4 cycles) for Nb/BDD and Nb/BDD-Ag electrodes.

Electrodeposited silver led to several changes in the Raman spectra with respect to the original Nb/BDD (Fig. 4a), where a reduction in the intensity of the boron-indicating and sp^3^ peaks was observed, coupled with higher intensity signal at approximately 1600 cm^−1^ due to the presence of silver particles, which may provide a peak located in this area^72^ and/or enhance the G-band intensity.^73^ Nonetheless, the lack of a distinct silver peak impeded its quantitative detection through Raman spectroscopy. To calculate the active surface area, CV experiments were conducted (Fig. 4b). It was observed that the ferrocyanide oxidation peak exhibited a significantly larger height in contrast to Nb/BDD, although it was reduced with successive oxidation–reduction cycles. The active surface area and mass transfer coefficient of Nb/BDD-Ag were calculated based on the highest peak value (Table B.1, ESI†), leading to a maximum active area of 170.1 cm^2^. Given the instability of the cyclic voltammograms in the presence of silver, degradation experiments with the three electrodes were defined in terms of the absolute current applied instead of the current density, as otherwise a continuous monitoring system of the active surface area would need to be in place.

**Fig. 5:**
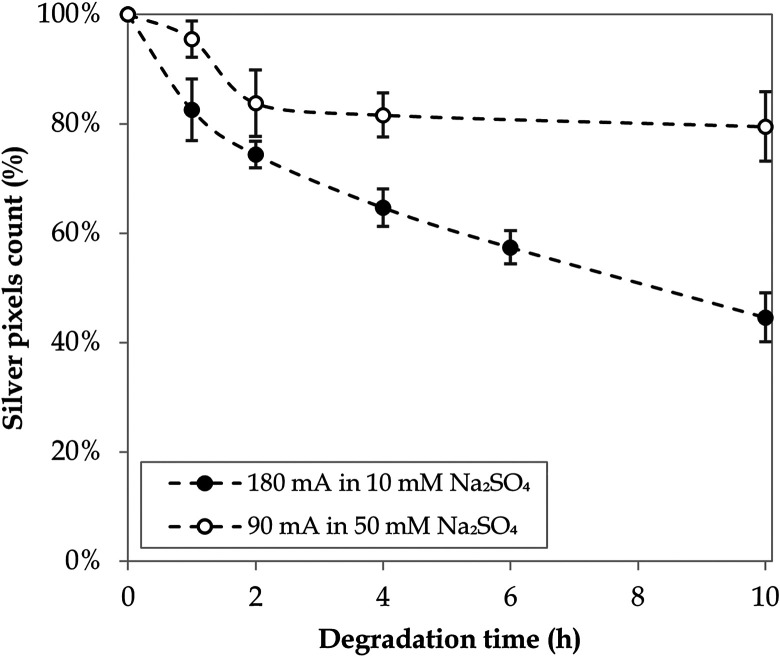
Stability tests of the Nb/BDD-Ag electrode surface.

To investigate the lifetime of the electrodeposited silver particles, stability tests were conducted under the experimental conditions later used in the Taguchi experiments, that is, 90 mA in 50 mM Na_2_SO_4_ and 180 mA in 10 mM Na_2_SO_4_. Each stability test was conducted for a period of 10 h, during which the electrode surface was examined *via* SEM at specific time intervals (Fig. B.3 and B.4, ESI†). In both cases, the silver particles showcased a transformation from a micro-flower shape into geometric structures that further detached or broke down into smaller pieces, potentially following a pulverisation effect.^74^ Blank experiments in the absence of electric current confirmed that no significant natural dissolution of silver into the anolyte took place, regardless of the sulfate concentration (Fig. B.5, ESI†). The stability of the Nb/BDD-Ag surface over time was quantified in terms of the reduction in the count of saturated pixels corresponding to silver deposits (Fig. 5) and in terms of the evolution of the particle size distributions (Fig. B.6, ESI†). After a first significant decrease in the pixel count of silver particles, operating at a lower current led to a plateau of approximately 80–84% of the initial silver still available between 2 and 10 h of degradation (Fig. 5). Similarly, the particle size distribution was stable after the first hour of operation (Fig. B.6a, ESI†). On the other hand, a higher current caused a continuous elimination of silver deposits, where 83% of the initial pixel count was already attained after 1 h and only 45% was left after 10 h (Fig. 5). The size reduction of the silver particles was also more drastic under these conditions, as reflected by the evolution of the particle size distributions (Fig. B.6b, ESI†).

### 3.3 Distribution of degradation mechanisms: influence of operating conditions on a commercial BDD

**Fig. 6:**
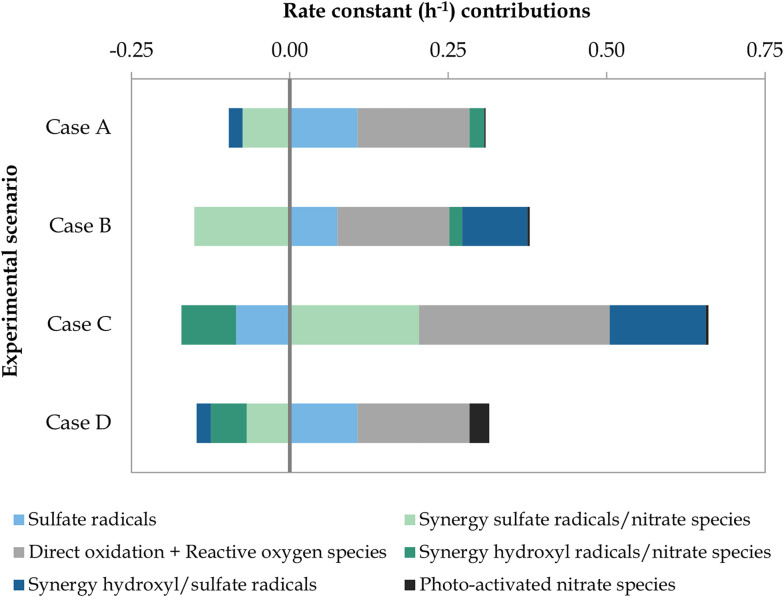
Rate constant contributions (h^−1^) of the degradation mechanisms involved in the removal of BA through scenarios A to D. The operating parameters of each experimental scenario are depicted in Table 2.

After implementing the model defined in Table A.1, ESI† to the scenarios presented in Table 2, the contributions of the different degradation mechanisms involved in the removal of BA with the commercial Nb/BDD electrode were calculated (Fig. 6). Underlying data can be found in Fig. C.1 and Tables C.1 and C.2, ESI,† and other experimental observations are presented in Table C.3, ESI.†

When comparing scenarios A and B, that is, when increasing the SO_4_^2−^ content of the wastewater, the contribution of the synergistic effect involving SO_4_˙^−^ and ˙OH radicals was significantly enhanced by 562%, transforming it from a negative to a positive value. The contribution by SO_4_˙^−^ radicals alone was hindered by 30%, which could be explained by an enhanced recombination to form S_2_O_8_^2−^ (eqn (11))^12–18,23^ and/or predominant scavenging reactions (eqn (4), (5), (22), (23) and (26)).^12,13,16,18,22,37^ Additional reactions forming S_2_O_8_^2−^ (eqn (10) and (12))^13,15–17,23^ could also be justified by the observed 26% increase in electrogenerated S_2_O_8_^2−^. Therefore, it could be argued that with increasing SO_4_^2−^ concentration, the transformation of SO_4_^2−^ into SO_4_˙^−^*via* ˙OH radicals was promoted (eqn (3))^12,14–18^ and *vice versa* (eqn (4) and (5)),^12,13,16,18,22^ as well as the subsequent radical interactions (eqn (6)–(9) and (12)–(16))^13,14,17,18,22,23^ included in their synergistic effect. In contrast, higher SO_4_^2−^ to NO_3_^−^ ratio led to a hindering effect in the synergies of NO_3_^−^ with both SO_4_˙^−^ and ˙OH (*i.e.*, reduced by 103% and 10%, respectively). It could be argued that SO_4_^2−^ was more easily adsorbed onto the electrode surface and impeded the potential activation of NO_3_^−^, which acted as a scavenger of ˙OH (eqn (34)) and SO_4_˙^−^ (eqn (35)).^13,18,39^ Photodegradation due to NO_3_^−^ (ref. 75) was negligible in both scenarios (*i.e.*, less than 2% contribution to the overall degradation). Overall, the degradation rate constant of BA slightly increased, from 0.212 to 0.228 h^−1^. In both cases A and B, the degradation was mainly driven by direct oxidation at the anode and ROS, which was not affected by the change in experimental conditions, followed by the contribution of the individual SO_4_˙^−^-driven mechanisms. In scenario B, the synergistic effect of SO_4_˙^−^ and ˙OH radicals was also a key promoting mechanism during the oxidation of BA (*i.e.*, 45% contribution).

Regarding the influence of the current applied, its increase between scenarios B and C led to a considerable enhancement of the BA degradation kinetic constant, from 0.228 to 0.490 h^−1^. The higher current promoted all synergistic effects involving SO_4_˙^−^ radicals, with a 48% and 235% increase in the synergies with ˙OH and NO_3_^−^, respectively. The latter increase could be due to the possible inhibition of the reaction described in eqn (35)^13,18,39^ and/or the promotion of nitrate-related oxidative species. As a result, SO_4_˙^−^-based synergies acquired a more relevant role in the degradation of BA. The contribution of direct anodic oxidation and ROS was increased by 70%, making it again the leading degradation mechanism. On the other hand, the SO_4_˙^−^ radical contribution was diminished by 213%, transforming it into a negative value. This decrease could be again related to the observed 74% increase in electrogenerated S_2_O_8_^2−^ (Table C.3, ESI†) resulting from an enhanced recombination of SO_4_˙^−^ (eqn (11)),^12–18,23^ as well as relevant scavenging reactions (eqn (4), (5), (10), (22), (23) and (26)).^12,13,15,16,18,22,37^ The synergy between ˙OH and NO_3_^−^ was considerably aggravated by 520%. This could be due to a reduced ˙OH efficiency resulting from the higher applied potential and subsequently promoted OER together with eqn (24) and (25),^22,37^ as suggested by the 90% increase in electrogenerated H_2_O_2_. Finally, for scenario C, photodegradation effects from NO_3_^−^ corresponded to less than 1% of the BA degradation.

Increasing the NO_3_^−^ concentration between scenarios A and D turned out to be overall unfavourable to BA degradation, as shown by the lowest kinetic constant observed (*i.e.*, 0.168 h^−1^). While most contributors remained constant, the synergistic effect of ˙OH and NO_3_^−^ was significantly aggravated by 346%, which augmented its negative contribution. The synergy between SO_4_˙^−^ and NO_3_^−^ exhibited a minor increase (*i.e.*, 8%) with regard to that of scenario A. Given the higher NO_3_^−^ content, photodegradation-driven mechanisms were promoted, representing 18% of the overall BA degradation. Similar to scenarios A and B, the main degradation drivers were direct oxidation and ROS as well as individual SO_4_˙^−^-driven mechanisms.

Finally, it should be noted that *in situ* formation of H_2_O_2_ was minor in all scenarios (Table C.3, ESI†) considering the poor ability of the stainless steel cathode to reduce O_2_. Therefore, an insignificant amount of ˙OH radicals can be expected *via* this route.^76^ The opposite could have been observed if platinum or graphite were used as cathode materials.^76^ For both electrogenerated H_2_O_2_ and S_2_O_8_^2−^, BA degradation by them alone was negligible (Table C.1, ESI†), although this does not rule out their participation in other reactions as described above. For the pH evolution during treatment, the pH increased by approximately 2 units in the four scenarios (Table C.3, ESI†), possibly explained by the formation of OH^−^ ions in eqn (3)^12,14–18^ and/or (13).^18,22^

### 3.4 Distribution of degradation mechanisms: multivariate optimisation *via* the Taguchi method

**Fig. 7:**
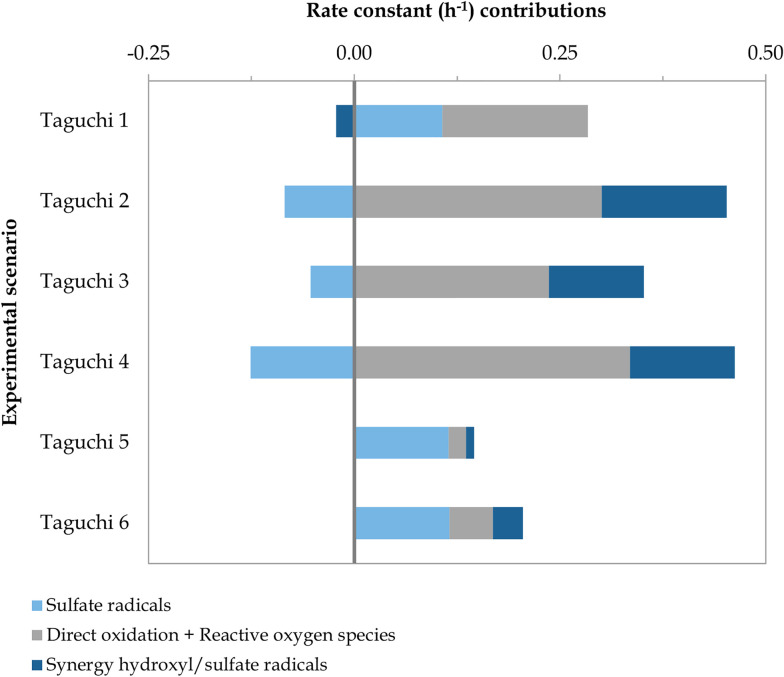
Rate constant contributions (h^−1^) of the degradation mechanisms involved in the removal of BA under the Taguchi design. The operating parameters of each experimental scenario are depicted in Table 3.

The Taguchi method was applied to conduct a multivariate optimisation study entailing diverse experimental parameters (*i.e.*, type of BDD, initial sulfate concentration, and applied current). Several optimisation targets were set and analysed separately, aimed at maximising the contribution of different degradation mechanisms (*i.e.*, SO_4_˙^−^, synergistic effect of SO_4_˙^−^ and ˙OH, and direct oxidation and ROS) as well as the overall BA degradation rate constant. The input values for the optimisation study were the rate constant contributions obtained when implementing the model defined in Table A.2, ESI† to the scenarios included in Table 3. These contributions are depicted in Fig. 7. Underlying data can be found in Fig. D.1 and Table D.1, ESI,† and other experimental observations (*e.g.*, pH, initial COD, H_2_O_2_ and S_2_O_8_^2−^ generation) are presented in Table D.2, ESI.†

As shown in Fig. 7, there was a significant shift in the distribution of degradation mechanisms when the Nb/BDD-Ag electrode was used, corresponding to scenarios Taguchi 5 and 6, in contrast to Nb/BDD (*i.e.*, Taguchi 1 and 2) and Si/BDD (*i.e.*, Taguchi 3 and 4). Regardless of the current applied and the initial SO_4_^2−^ concentration, BA degradation was driven by SO_4_˙^−^-driven mechanisms in both Taguchi 5 and 6. This could be attributed to the catalyst role of the silver microparticles electrodeposited on the BDD surface, as they are reactive towards electrogenerated S_2_O_8_^2−^ (eqn (18) and (19))^16,26^ and H_2_O_2_ (eqn (20))^33^ to form SO_4_˙^−^ and ˙OH radicals, respectively. In fact, *in situ* formation of S_2_O_8_^2−^ and H_2_O_2_ was not detected, as opposed to the scenarios with commercial BDD electrodes (Table D.2, ESI†). On the other hand, the contribution of direct oxidation and ROS was dramatically reduced, possibly due to the silver deposits affecting the electroactive surface area. Another distinctive feature was the minor change in pH in both Taguchi 5 and 6 (Table D.2, ESI†), which may be associated with the decrease in the synergistic effect of SO_4_˙^−^ and ˙OH radicals, and hence, to the inhibition of reactions forming OH^−^ ions (eqn (3) and (13)).^12,14–18,22^ The influence of the electrode type can be compared in absolute terms between scenarios Taguchi 4 and 6 and Taguchi 3 and 5, as the same current and initial SO_4_^2−^ concentration were used. In these comparative scenarios, Nb/BDD-Ag led to a 71–92% decrease in synergistic effects as well as an 84–91% reduction in direct oxidation and ROS contributions with respect to the Si/BDD electrode. The contribution of SO_4_˙^−^-driven mechanisms exhibited a 316% and 192% increase in experiments Taguchi 5 and 6, respectively.

It is also possible to compare the effects of simultaneously increasing the initial SO_4_^2−^ concentration and current applied between scenarios Taguchi 1 and 2, where the electrode used was Nb/BDD. The contribution of the synergistic effect of SO_4_˙^−^ and ˙OH radicals was enhanced by 786%, going from a negative to a positive value, and that of direct oxidation and ROS was increased by 70%. As previously observed between scenarios A, B, and C, the increased current and initial SO_4_^2−^ concentration are key drivers for these effects. In contrast, reactions involving only SO_4_˙^−^ radicals were hindered by 179%, transforming it into a negative contributor. The reduction in SO_4_˙^−^-driven mechanisms could again be explained by the increased initial SO_4_^2−^ triggering the recombination of SO_4_˙^−^ (eqn (11))^12–18,23^ and/or its self-recombination (eqn (10))^13,15–17^ since a 277% increase in electrogenerated S_2_O_8_^2−^ was observed, and/or other scavenging reactions (eqn (4), (5), (22), (23) and (26)).^12,13,16,18,22,37^ Therefore, increasing both the current and initial SO_4_^2−^ concentration seems to again promote the transformation of SO_4_^2−^ into SO_4_˙^−^*via* ˙OH radicals (eqn (3))^12,14–18^ and *vice versa* (eqn (4) and (5)),^12,13,16,18,22^ as well as the subsequent synergistic effects (eqn (6)–(9) and (12)–(16)).^13,14,17,18,22,23^ In terms of BA degradation, its kinetic constant was considerably enhanced from 0.262 h^−1^ in Taguchi 1 to 0.368 h^−1^ in Taguchi 2.

**Fig. 8:**
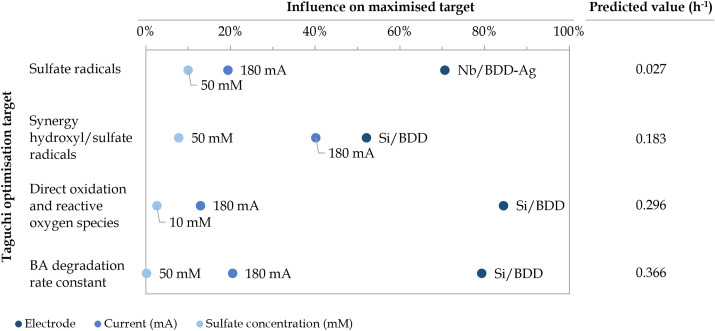
Influence of the optimal experimental conditions (*i.e.*, electrode type, current applied, and initial sulfate concentration) on the selected Taguchi optimisation targets, that is, aiming to maximise the degradation *via* sulfate radicals, sulfate/hydroxyl synergistic effect, direct oxidation and ROS as well as the overall BA degradation rate constant. Predicted contributions (h^−1^) under the optimal conditions are also provided.

Based on the distribution of the different degradation mechanisms in the removal of BA (Fig. 7), the optimal value of each experimental parameter (*i.e.*, the value that maximises the selected optimisation target) was identified based on average signal-to-noise ratio (S/N) values (Fig. D.2, ESI†), as defined by Taguchi.^49^ In addition, the influence of each parameter on the maximised target was estimated through ANOVA (Table D.3, ESI†).^49^ A summary of the optimal values, their influence and the predicted result under optimal conditions are depicted in Fig. 8.

As shown in Fig. 8, the experimental parameter that presents the highest influence on all optimisation targets is the nature of the electrode used, accounting for approximately 52–85% of the attained results, followed by the current applied (*i.e.*, 13–40% influence) and the initial SO_4_^2−^ concentration (*i.e.*, 0.2–10% influence). When aiming at maximising the contribution of SO_4_˙^−^-driven mechanisms individually, Nb/BDD-Ag was found to be the optimal electrode type, as already inferred from Fig. 7, presumably due to the catalyst role of the silver electrodeposited microparticles (eqn (18)–(20)).^16,26,33^ With this electrode, a higher current (*i.e.*, 180 mA) and higher initial SO_4_^2−^ concentration (*i.e.*, 50 mM) were the optimal conditions to increase the degradation *via* SO_4_˙^−^ alone, up to a predicted 0.027 h^−1^ rate constant. This may be due to the associated rise in S_2_O_8_^2−^ when increasing both experimental parameters, leading to its further reaction with silver.

On the other hand, the Si/BDD electrode, which had a lower sp^3^ content due to higher boron doping, was the optimal electrode to maximise the contribution of direct oxidation and ROS up to a predicted rate constant of 0.296 h^−1^ as well as the synergistic effect of SO_4_˙^−^ and ˙OH radicals to an estimated value of 0.183 h^−1^. Its higher non-diamond content (*e.g.*, boron-related defects and graphitic carbon impurities) corresponds to an “active electrode”, which facilitates adsorption and desorption mechanisms, that is, electrochemical conversion rather than electrochemical combustion.^13,77^ Moreover, under high sp^2^ levels, graphitic carbon is oxidised into CO_2_ by ˙OH radicals, reducing the electrochemical activity.^13^ The Nb/BDD electrode was not suitable for these optimisation targets because it presented a lower boron content and slightly larger grains (consequently, higher sp^3^ content), which is known to contribute to the production of ˙OH radicals as it behaves as a “non-active anode”.^77^ The use of niobium as a substrate material was also reported to contribute to ˙OH formation.^78^ Nonetheless, there was no dramatic difference in the average S/N between Si/BDD and Nb/BDD when maximising direct oxidation and ROS contributions. This could be explained by the fact that these were calculated as a whole and that, even if different electrodes may favour opposite mechanism, the sum of both contributions eventually remained similar.

Given that a higher sp^2^ content in Si/BDD was also reported to enhance adsorption sites for SO_4_^2−^ and favour SO_4_˙^−^ radical formation,^79^ a lower initial SO_4_^2−^ concentration (*i.e.*, 10 mM) was preferred to promote the contribution of direct oxidation and ROS. Conversely, a higher initial SO_4_^2−^ concentration (*i.e.*, 50 mM), which also led to an enhanced formation of S_2_O_8_^2−^, was found to promote the synergistic effect of SO_4_˙^−^ and ˙OH radicals. Nevertheless, there was again no significant difference in the average S/N between these initial SO_4_^2−^ concentrations, which, combined with their lower influence on the maximised targets, demonstrated the reduced impact of this process variable. Regarding the optimal current, applying a higher value (*i.e.*, 180 mA) was the second most influential factor to both maximisation targets, likely motivated by the increased electron flow. The overall BA degradation rate constant was estimated to achieve a maximum rate constant of 0.366 h^−1^ under the same electrode and current conditions as the maximised direct oxidation and ROS and synergistic effects scenarios, given that both are the main contributors to its degradation.

## 4 Evaluation of the approach

Our experimental and computational approach represents a straightforward and simple method to estimate the distribution of degradation mechanisms without requiring advanced techniques. In addition, it goes beyond identifying single radical species by considering a broader range of radical and non-radical mechanisms as well as their synergies. Regarding its implementation, our approach is however limited to sulfate- and nitrate-containing effluents. Therefore, future research should be dedicated to the inclusion of other electrogenerated species, such as chlorine radicals (Cl˙) and nitrite radicals (NO_2_˙), which are potentially formed when chloride (Cl^−^) and nitrite (NO_2_^−^) ions are present in the influent wastewater, respectively.^80^ In addition, the model would benefit from the development of new strategies to differentiate between direct oxidation and ROS contributions as well as to identify and quantify the contribution of ˙OH radicals and nitrate-related oxidative species individually. To this end, EPR analyses, quantum chemical calculations or the elucidation of more specific scavenging compounds would enable the consolidation and extension of the kinetic model proposed.

The accuracy of the approach can be evaluated through the optimal conditions that maximise the contribution of the synergistic effect between SO_4_˙^−^ and ˙OH radicals and the overall BA degradation rate constant (Fig. 8), as they correspond to the fourth Taguchi scenario carried out (Table 3). Regarding the synergistic effect, the predicted contribution (*i.e.*, 0.183 h^−1^) was 44% higher than the observed contribution (*i.e.*, 0.127 h^−1^), whereas the predicted BA degradation rate constant (*i.e.*, 0.366 h^−1^) was 9% higher than the experimental value (*i.e.*, 0.337 h^−1^). These overestimations in the model could be mitigated by an increase in experimental replicates so that standard deviations could be considered for each contributor in the distribution of degradation mechanisms. As a result, ANOVA could also account for errors, and a confidence interval for the predicted value could be provided.

Regarding the attained results, an open question regards the influence of other electrode characteristics, such as roughness and morphology. In this research, these were differentiating properties between the electrodes, although the influence of each individually needs to be further evaluated. Similarly, the modification of the BDD electrode surface with silver microparticles allowed to demonstrate the effects of metal catalysts on the degradation mechanisms, although the applied decoration route is not sustainable in the long term, as evidenced by the stability tests. Further research could be focused on evaluating other metal catalysts, refining the surface decoration method, and maximising the catalyst lifetime and regeneration. As a result, it would be possible to accurately evaluate the feasibility of this type of treatment with respect to previous BA degradation studies (Table E.1, ESI†).

## 5 Conclusions

Given the need for advanced technical tools, the identification and quantification of the degradation mechanisms involved in eAOPs are arduous activities to implement in real scenarios. To overcome this limitation and shed light on the fundamentals behind electrochemical wastewater treatment, a combined experimental and computational approach was proposed to estimate the contribution of radical and non-radical mechanisms as well as their synergistic effects in sulfate- and nitrate-containing effluents treated with BDD anodes.

When applying our combined experimental and computational approach to the degradation of BA, it was found that its removal was mainly driven by direct oxidation at the anode and ROS, followed by radical synergistic effects. These mechanisms were also enhanced with increasing SO_4_^2−^ concentration and current applied. By visualising the distribution of degradation mechanisms, it was also possible to identify the negative effects derived from NO_3_^−^ ions and their response to changes in operating parameters. Our approach also provided insights to enable further process optimisation, as degradation mechanisms are strongly dependent on factors such as electrode type, wastewater composition, current density, and the presence of metal catalysts. To this end, a multivariate optimisation study was conducted, which allowed for the elucidation of the influence of the initial SO_4_^2−^ concentration, current applied, and electrode characteristics of two commercial BDD electrodes and a customised silver-decorated BDD electrode. The results showed that the nature of the electrode was the most relevant factor to the attained efficiency, given that properties such as the boron content in the BDD material and the presence of electrodeposited silver could dramatically affect the reactions taking place. Depending on the degradation mechanisms to be favoured, different optimal operating conditions were identified. In the case of maximising direct oxidation and ROS, the use of a BDD with higher boron content on a silicon substrate in combination with higher current and lower SO_4_^2−^ concentration was optimal, whereas enhancing synergistic effects would primarily be achieved by implementing these conditions with a higher initial SO_4_^2−^ concentration. Conversely, decorating the BDD surface with silver microparticles significantly enhanced the degradation mechanisms entailing SO_4_˙^−^ radicals individually and hindered other degradation routes.

In summary, this combined experimental and computational approach is a good starting point to understand and optimise electrochemical wastewater treatment applications without advanced analytical tools. Nonetheless, future research is needed to broaden the kinetic model proposed so that our approach becomes more versatile and applicable to wastewater effluents of miscellaneous compositions. In addition, this work briefly explored promising opportunities for BDD surface decoration with silver microparticles to promote radical-driven mechanisms. Nonetheless, an in-depth electrocatalysis study is required to optimise the electrodeposition method as well as to assess its feasibility for future applications.

## Abbreviations

AFM: Atomic force microscope
AOP(s): Advanced oxidation process(es)
BA: Benzoic acid
BDD: Boron-doped diamond
CEC(s): Contaminant(s) of emerging concern
COD: Chemical oxygen demand
CV: Cyclic voltammetry
eAOP(s): Electrochemical advanced oxidation process(es)
EDS: Energy dispersive X-ray spectrometer
EPR: Electron paramagnetic resonance
NB: Nitrobenzene
OER: Oxygen evolution reaction
PMS: Peroxymonosulfate
PS: Persulfate
RMS: Root mean square
ROS: Reactive oxygen species
SEM: Scanning electron microscope
S/N: Signal-to-noise ratio
(U)HPLC: (Ultra-)high performance liquid chromatography

## Author contributions

S. Feijoo: conceptualisation, methodology, software, validation, formal analysis, investigation, writing – original draft, writing – review & editing, visualisation. S. Baluchová: resources, writing – review & editing, supervision. M. Kamali: conceptualisation, writing – review & editing, supervision. J. G. Buijnsters: resources, writing – review & editing, supervision, funding acquisition. R. Dewil: conceptualisation, resources, writing – review & editing, supervision, project administration, funding acquisition.

## Conflicts of interest

There are no conflicts to declare.

## Supplementary Material

EW-010-D3EW00784G-s001
