# Supplementary material for: A combined experimental and computational approach to unravel degradation mechanisms in electrochemical wastewater treatment
Source: Environ Sci (Camb). 2024 Jan 19;10(3):652–67. doi: 10.1039/d3ew00784g (PMC10905665; doi:10.1039/d3ew00784g)
Supplement: EW-010-D3EW00784G-s001 [file EW-010-D3EW00784G-s001.pdf]

Electronic Supplementary Material (ESI) for Environmental Science: Water Research & Technology.
This journal is © The Royal Society of Chemistry 2024

A combined experimental and computational approach to unravel degradation mechanisms in electrochemical wastewater treatment

Sara Feijoo[1], Simona Baluchová[2], Mohammadreza Kamali[1], Josephus G. Buijnsters[2], and Raf Dewil[1,3,*]

[1]KU Leuven, Department of Chemical Engineering, Process and Environmental Technology Lab, Jan Pieter de Nayerlaan 5, 2860 Sint-Katelijne-Waver, Belgium

[2]Delft University of Technology, Department of Precision and Microsystems Engineering, Mekelweg 2, 2628 CD Delft, The Netherlands

[3]University of Oxford, Department of Engineering Science, Parks Road, Oxford, OX1 3PJ, United Kingdom

[*]Corresponding author: Raf Dewil, raf.dewil@kuleuven.be

Supplementary Material

A Kinetic model & Design of experiments

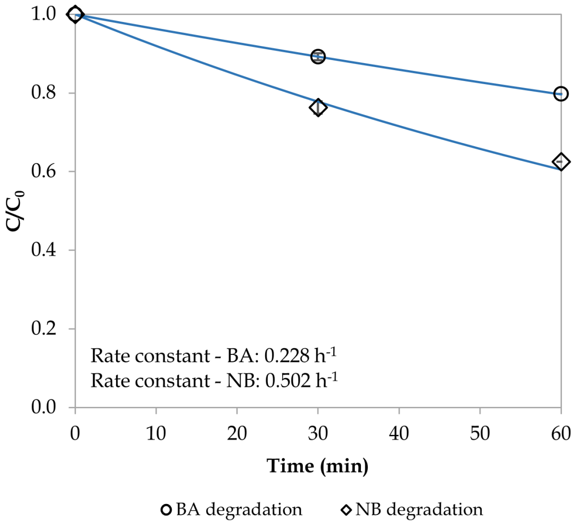

Figure A.1: Simultaneous degradation of 10 μM BA and NB in 10 mM Na_2SO_4 solution at 0.09 A.

Table A.1: Simplified experimental and computational approach to unravel the distribution of degradation mechanisms in scenarios A to D.

Exp.	Operating conditions	Model implementation	Calculated parameter
3	BA photodegradation in $SO_4{}^{2-}$ and $NO_3{}^{-}$ medium (no current applied).	$k_{BA,3} = \mid \lambda_{NO_3^-} \mid$	$\mid \lambda_{NO_3^-} \mid$
4	Electrogeneration of H_2O_2 and $S_2O_8{}^{2-}$ in $SO_4{}^{2-}$ and $NO_3{}^{-}$ medium without BA (current applied).	Not applicable	$[H_2O_2]$, $[S_2O_8{}^{2-}]$
8	BA degradation in pure water with methanol as scavenger for $^{\bullet}OH$ radicals (current applied). Sodium acetate was added to increase the conductivity.	$k_{BA,8} = \mid A_{ox} \mid + \mid ROS \mid$	$\mid A_{ox} \mid + \mid ROS \mid$
11	BA degradation in $SO_4{}^{2-}$ medium with tert-butanol as scavenger for $^{\bullet}OH$ radicals (current applied).	$k_{BA,11} = \mid SO_4^{\bullet -} \mid + \mid A_{ox} \mid + \mid ROS \mid$	$\mid SO_4^{\bullet -} \mid$
12	BA degradation in $SO_4{}^{2-}$ medium (current applied).	$k_{BA,12} = \mid SO_4^{\bullet -} \mid + \mid A_{ox} \mid + \mid ROS \mid + \mid S_{^{\bullet}OH,SO_4^{\bullet -}} \mid$	$\mid S_{^{\bullet}OH,SO_4^{\bullet -}} \mid$
13	BA degradation in $NO_3{}^{-}$ medium (current applied).	$k_{BA,13} = \mid A_{ox} \mid + \mid ROS \mid + \mid \lambda_{NO_3^-} \mid + \mid S_{^{\bullet}OH,NO_3^-} \mid$	$\mid S_{^{\bullet}OH,NO_3^-} \mid$
Target	BA degradation in $SO_4{}^{2-}$ and $NO_3{}^{-}$ medium (current applied).	$k_{obs,BA} = \mid SO_4^{\bullet -} \mid + \mid A_{ox} \mid + \mid ROS \mid + \mid \lambda_{NO_3^-} \mid +$ $+ \mid S_{^{\bullet}OH,SO_4^{\bullet -}} \mid + \mid S_{^{\bullet}OH,NO_3^-} \mid + \mid S_{SO_4^{\bullet -},NO_3^-} \mid$	$\mid S_{SO_4^{\bullet -},NO_3^-} \mid$

Table A.2: Simplified experimental and computational approach to unravel the distribution of degradation mechanisms under the Taguchi design.

Exp.	Operating conditions	Model implementation	Calculated parameter
4	Electrogeneration of H_2O_2 and $S_2O_8{}^{2-}$ in $SO_4{}^{2-}$ medium without BA (current applied).	Not applicable	$[H_2O_2]$, $[S_2O_8{}^{2-}]$
8	BA degradation in pure water with methanol as scavenger for $^{\bullet}$OH radicals (current applied). Sodium acetate was added to increase the conductivity.	$k_{BA,8} =\mid A_{ox} \mid + \mid ROS \mid$	$\mid A_{ox} \mid + \mid ROS \mid$
11	BA degradation in $SO_4{}^{2-}$ medium with tert-butanol as scavenger for $^{\bullet}$OH radicals (current applied).	$k_{BA,11} =\mid SO_4^{\bullet-} \mid + \mid A_{ox} \mid + \mid ROS \mid$	$\mid SO_4^{\bullet-} \mid$
Target	BA degradation in $SO_4{}^{2-}$ medium (current applied).	$k_{obs,BA} =\mid SO_4^{\bullet-} \mid + \mid A_{ox} \mid + \mid ROS \mid + \mid S_{^{\bullet}OH,SO_4^{\bullet-}} \mid$	$\mid S_{^{\bullet}OH,SO_4^{\bullet-}} \mid$

B Electrode characterisation

Table B.1: Electrode characterisation results.

Electrode	Electroactive surface area (cm^2)	Mass transfer coefficient (k_m, m s^{-1})
Nb/BDD	57.1	$1.52 \cdot 10^{-5}$
Si/BDD	46.6	$1.52 \cdot 10^{-5}$
Nb/BDD-Ag	170.1	$1.52 \cdot 10^{-5}$

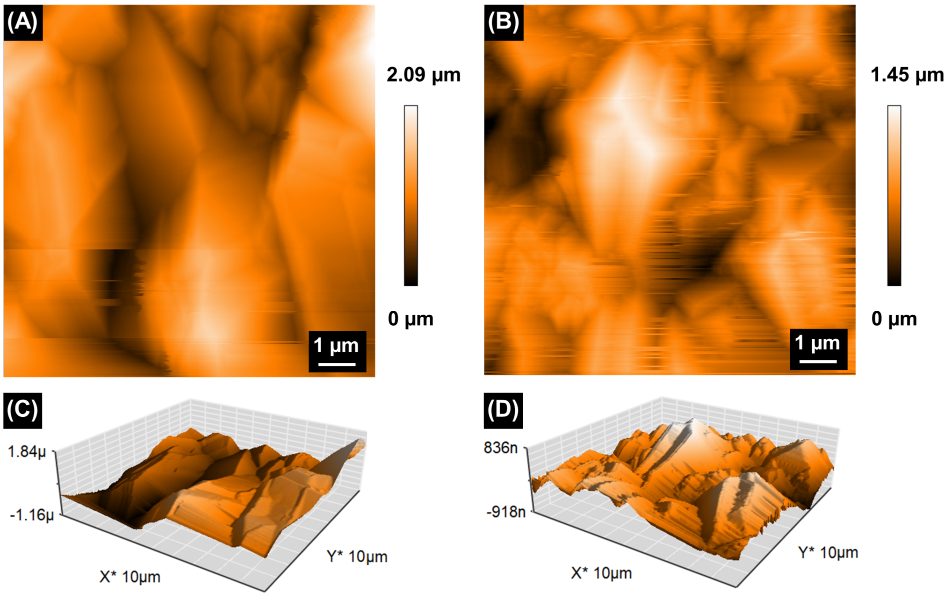

Figure B.1: AFM topographic and profile scans (10x10 μm^2) of commercial **(a)**,**(c)** Nb/BDD and **(b)**,**(d)** Si/BDD electrodes.

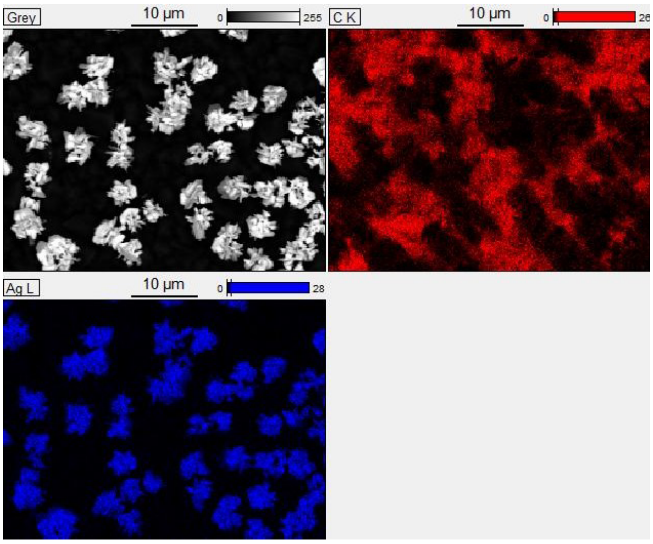

Figure B.2: Elemental mapping of the Nb/BDD-Ag electrode based on EDS analysis.

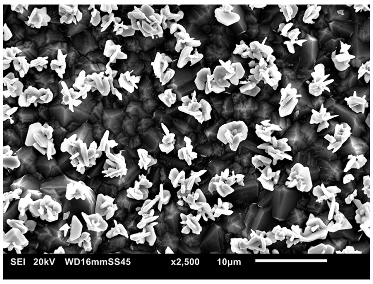

(a) $t = 0$ h.

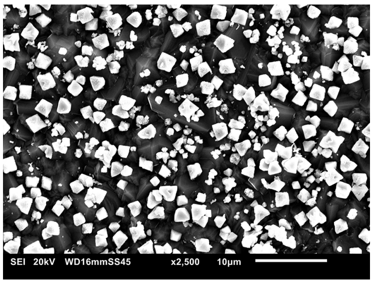

(b) $t = 1$ h.

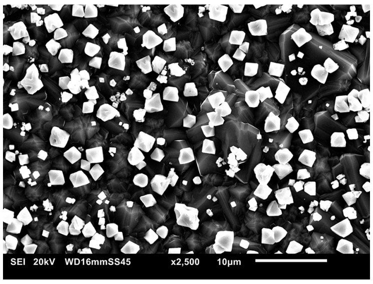

(c) $t = 2$ h.

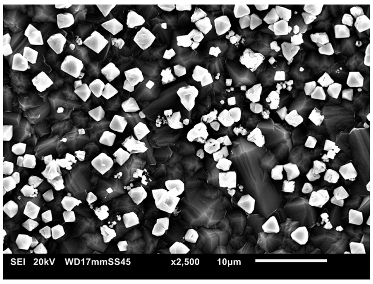

(d) $t = 4$ h.

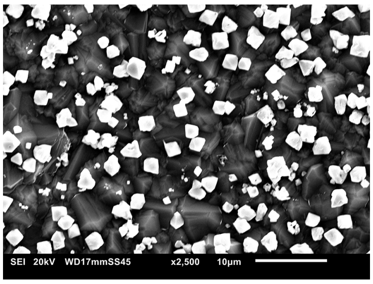

(e) $t = 10$ h.

Figure B.3: SEM images of electrodeposited Nb/BDD-Ag during a 10-h stability test at 90 mA in 50 mM Na_2SO_4.

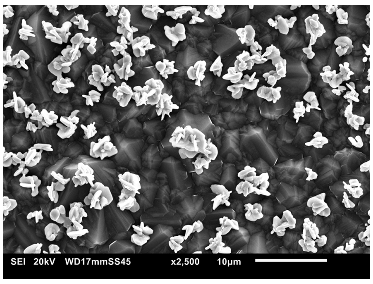

(a) $t = 0$ h.

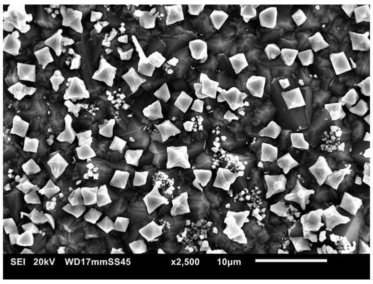

(b) $t = 1$ h.

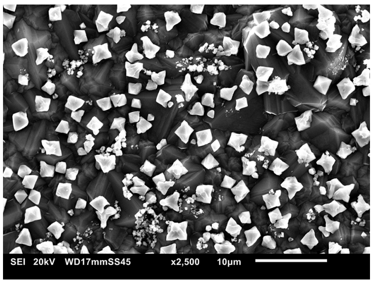

(c) $t = 2$ h.

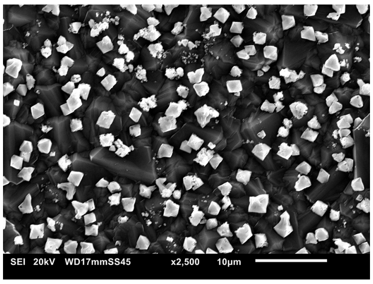

(d) $t = 4$ h.

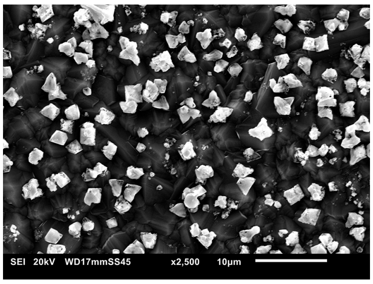

(e) $t = 6$ h.

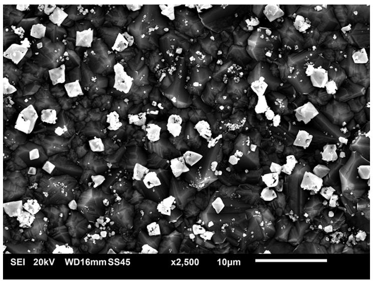

(f) $t = 10$ h.

Figure B.4: SEM images of electrodeposited Nb/BDD-Ag during a 10-h stability test at 180 mA in 10 mM Na_2SO_4.

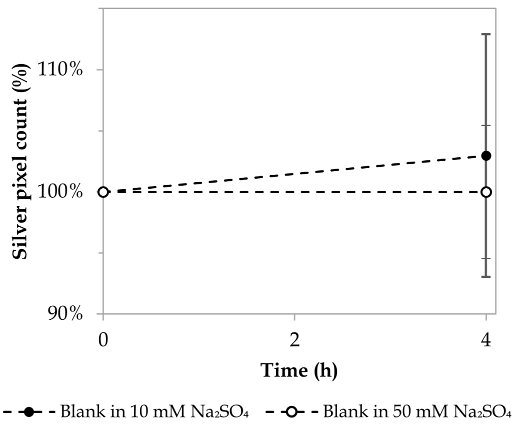

Figure B.5: Blank stability tests of a freshly prepared Nb/BDD-Ag electrode.

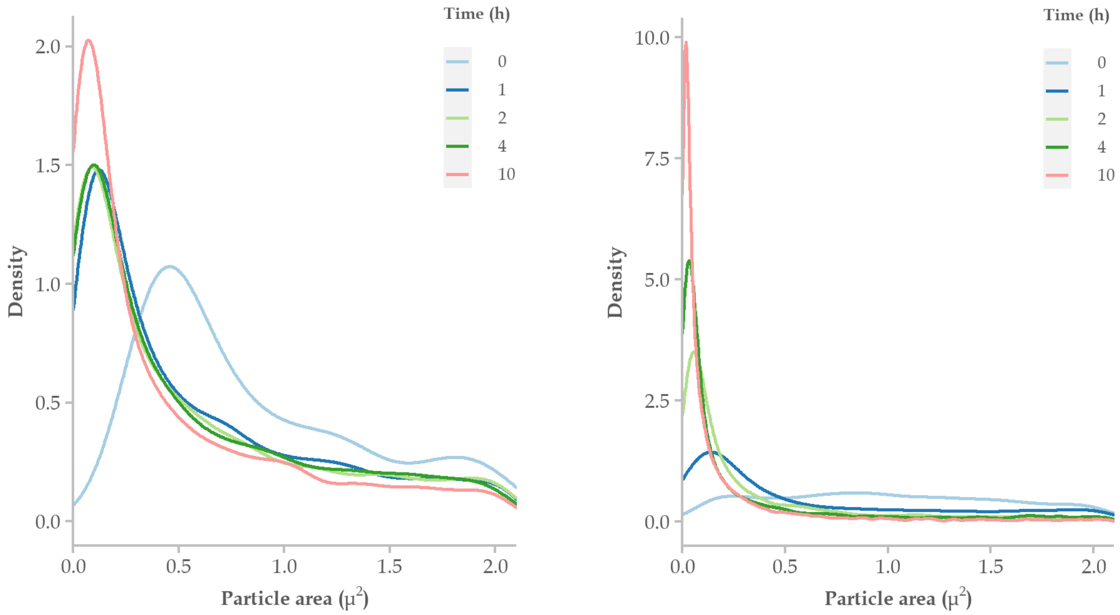

(a) **(b)**

Figure B.6: Silver particles density distribution during stability tests at **(a)** 90 mA in 50 mM Na_2SO_4 and **(b)** 180 mA in 10 mM Na_2SO_4.

C Distribution of degradation mechanisms: Influence of operating conditions on a commercial BDD

Table C.1: Insights into the BA degradation mechanisms through scenarios A to D.

Parameter	Rate constant (h^{-1})			
	Scenario A	Scenario B	Scenario C	Scenario D
$\|SO_4^{\bullet -}\|$	0.107	0.075	-0.084	0.107
$\|A_{ox}\| + \|ROS\|$	0.177	0.177	0.301	0.177
$\|S_{^{\bullet}OH,SO_4^{\bullet -}}\|$	-0.022	0.102	0.152	-0.022
$\|S_{SO_4^{\bullet -},NO_3^-}\|$	-0.074	-0.151	0.204	-0.068
$\|S_{^{\bullet}OH,NO_3^-}\|$	0.023	0.021	-0.087	-0.057
$\|\lambda_{NO_3^-}\|$	0.002	0.004	0.004	0.031
$\|S_2O_8^{2-}\|$	0.000	0.000	0.000	0.000
$\|H_2O_2\|$	0.000	0.000	0.000	0.000
$k_{obs,BA}$	0.212	0.228	0.490	0.168

Table C.2: Comparative analysis of scenarios A to D.

Cases	Change	$k_{obs,BA}$	$\|SO_4^{\bullet -}\|$	$\|A_{ox}\| + \|ROS\|$	$\|S_{^{\bullet}OH,SO_4^{\bullet -}}\|$	$\|S_{SO_4^{\bullet -},NO_3^-}\|$	$\|S_{^{\bullet}OH,NO_3^-}\|$
A & B	↑ SO_4^{2-}	+7%	-30%	≈	+562%	-103%	-10%
B & C	↑ Current	+115%	-213%	+70%	+48%	+235%	-520%
A & D	↑ NO_3^-	-21%	≈	≈	≈	+8%	-346%

Table C.3: Observed experimental results during the degradation of BA with the commercial Nb/BDD electrode.

Scenario	$k_{obs,BA}$ (h^{-1})	$S_2O_8^{2-}$ formation (mM)	H_2O_2 formation (mM)	pH change	Initial COD (mg O_2 L^{-1})	Limiting current (mA)
A	0.212	0.096 ± 0.006	0.084 ± 0.001	2.12 ± 0.38	5.97 ± 0.17	6.28
B	0.228	0.121 ± 0.003	0.108 ± 0.006	2.44 ± 0.37	6.43 ± 0.15	6.76
C	0.490	0.211 ± 0.006	0.205 ± 0.002	2.45 ± 0.05	6.43 ± 0.15	6.76
D	0.168	0.083 ± 0.001	0.074 ± 0.002	2.27 ± 0.41	7.79 ± 1.59	8.19

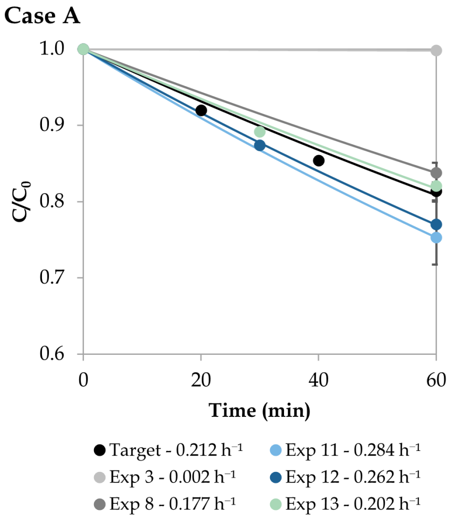

(a)

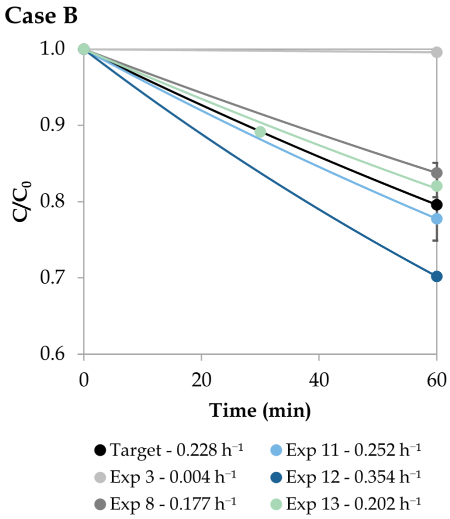

(b)

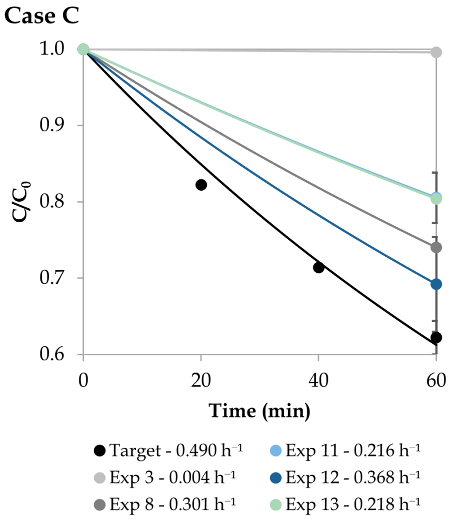

(c)

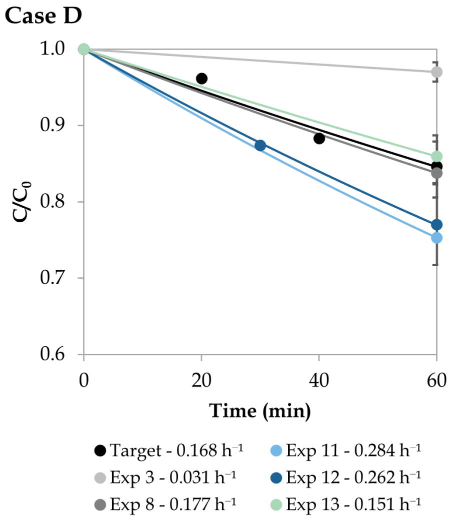

(d)

Figure C.1: Degradation profiles of BA in the experiments defined in Table A.1 for scenarios A to D.

D Distribution of degradation mechanisms: Taguchi optimisation

Table D.1: Insights into the BA degradation mechanisms under the Taguchi design.

Parameter	Rate constant (h^{-1})					
	**Taguchi 1**	**Taguchi 2**	**Taguchi 3**	**Taguchi 4**	**Taguchi 5**	**Taguchi 6**
$\lvert SO_4^{\bullet-}\rvert$	0.107	-0.084	-0.053	-0.126	0.115	0.116
$\lvert A_{ox}\rvert + \lvert ROS\rvert$	0.177	0.301	0.237	0.335	0.021	0.053
$\lvert S_{\bullet OH, SO_4^{\bullet-}}\rvert$	-0.022	0.152	0.115	0.127	0.010	0.036
$\lvert S_2O_8^{2-}\rvert$	0.000	0.000	0.000	0.000	0.000	0.000
$\lvert H_2O_2\rvert$	0.000	0.000	0.000	0.000	0.000	0.000
$k_{obs,BA}$	0.262	0.368	0.299	0.337	0.146	0.205

Table D.2: Observed experimental results during the degradation of BA under the Taguchi design.

**Taguchi test**	**$k_{obs,BA}$ (h^{-1})**	**$S_2O_8{}^{2-}$ formation (mM)**	**H_2O_2 formation (mM)**	**pH change**	**Initial COD (mg O_2 L^{-1})**	**Limiting current (mA)**
1	0.262	0.035 ± 0.001	0.025 ± 0.002	1.51 ± 0.30	8.07 ± 0.37	8.48
2	0.368	0.132 ± 0.002	0.108 ± 0.005	2.14 ± 0.37	9.59 ± 0.16	10.08
3	0.299	0.012 ± 0.004	0.005 ± 0.002	2.55 ± 0.46	9.59 ± 0.16	8.22
4	0.337	0.020 ± 0.003	0.015 ± 0.001	1.57 ± 0.33	8.07 ± 0.37	6.92
5	0.146	No detection	No detection	-0.58 ± 0.02	9.59 ± 0.16	29.99
6	0.205	No detection	No detection	0.92 ± 0.27	8.07 ± 0.37	25.24

Table D.3: Insights into the influence of the optimal experimental conditions (i.e., electrode type, current applied, and initial sulfate concentration) attained via ANOVA on the selected Taguchi optimisation targets, that is, aiming to maximise the degradation via sulfate radicals, synergy between hydroxyl and sulfate radicals, and direct oxidation and reactive oxygen species as well as the overall BA degradation rate constant.

**Parameter**	**DoF**	**Sum Sq**	**Mean Sq**	**Contribution**	**Optimum**
			Sulfate radicals		
Electrode type	2	0.042	0.021	70.6%	Nb/BDD-Ag
Current applied	1	0.012	0.012	19.4%	180 mA
Initial sulfate concentration	1	0.006	0.006	10.0%	50 mM
Total	4	0.059	-	100%	-
			Synergy hydroxyl/sulfate radicals		
Electrode type	2	0.010	0.005	52.1%	Si/BDD
Current applied	1	0.008	0.008	40.2%	180 mA
Initial sulfate concentration	1	0.001	0.001	7.8%	50 mM
Total	4	0.019	-	100%	-
			Direct oxidation and reactive oxygen species		
Electrode type	2	0.070	0.035	84.5%	Si/BDD
Current applied	1	0.011	0.011	12.9%	180 mA
Initial sulfate concentration	1	0.002	0.002	2.6%	10 mM
Total	4	0.083	-	100%	-
			BA degradation rate constant		
Electrode type	2	0.027	0.013	79.4%	Si/BDD
Current applied	1	0.007	0.007	20.5%	180 mA
Initial sulfate concentration	1	0.000	0.000	0.2%	50 mM
Total	4	0.033	-	100%	-

DoF: degrees of freedom, **Sum Sq:** sum of squares, **Mean Sq:** mean of squares.

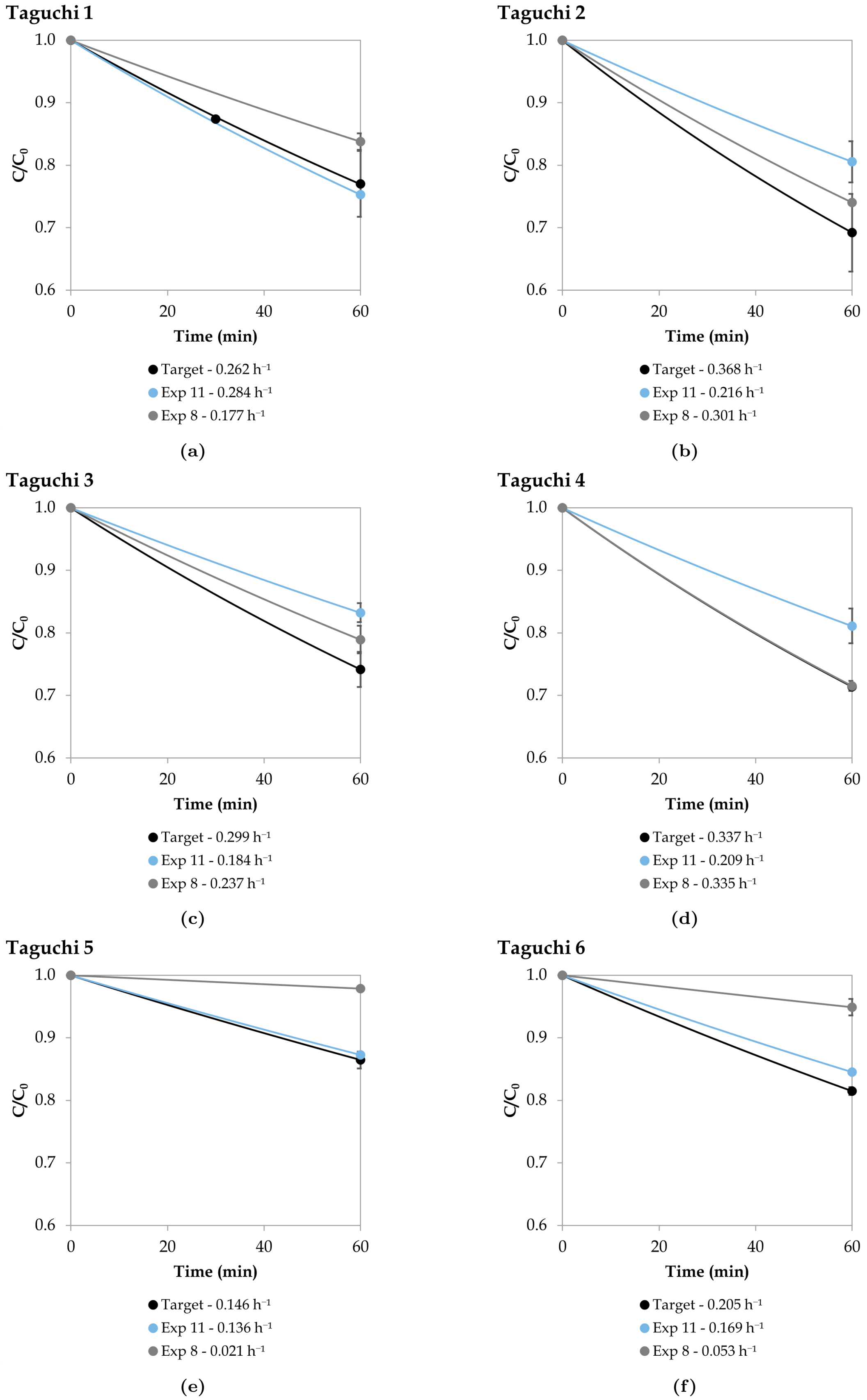

Figure D.1: Degradation profiles of BA in the experiments defined in Table A.2 under the Taguchi design.

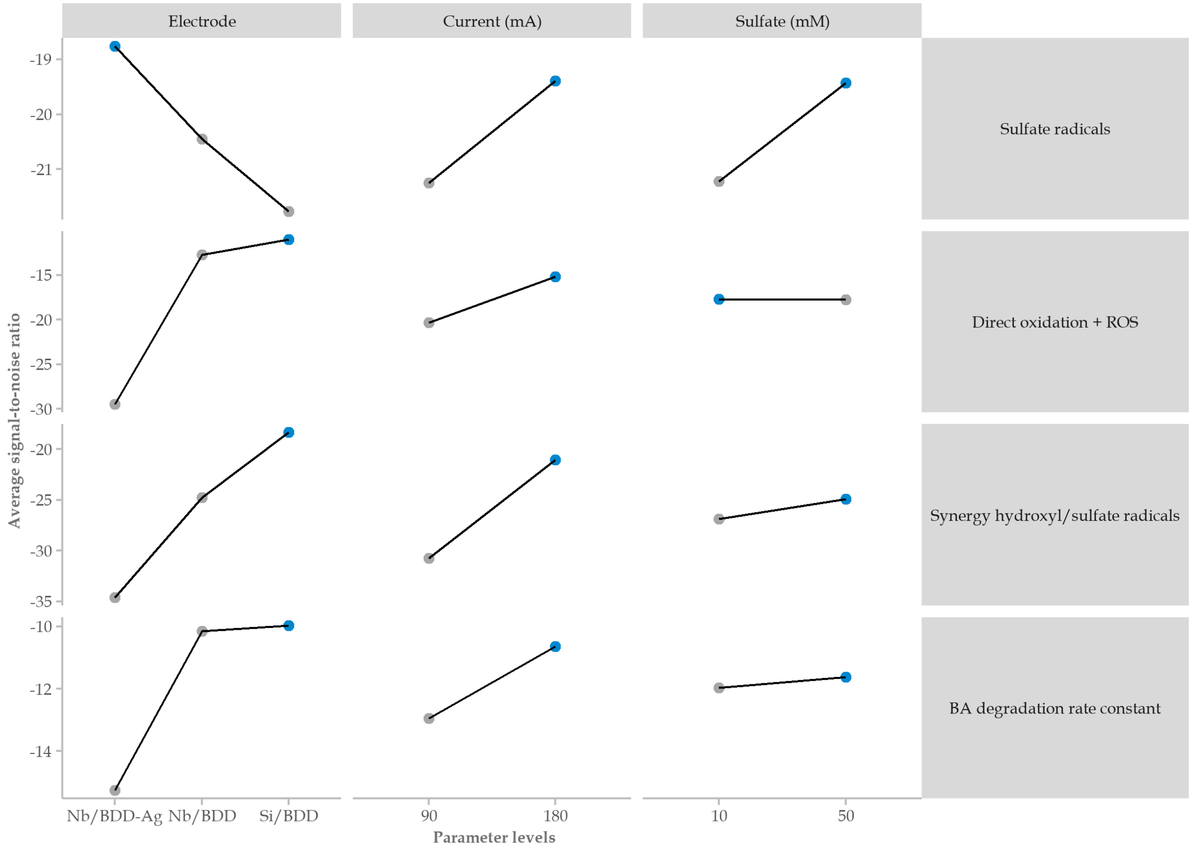

Figure D.2: Taguchi results when maximising the contribution of multiple degradation mechanisms (i.e., sulfate radicals, synergy between hydroxyl and sulfate radicals, and direct oxidation and reactive oxygen species) as well as the BA degradation rate constant. Optimum levels per parameter and maximisation target are highlighted in blue.

E Literature review

Table E.1: Comparison with previous studies on BA degradation.

Initial BA	Electrode	Total area (cm^2)	Anolyte composition	Electrical parameters	Degradation rate constant (h^{-1})	BA removed per hour ($mg\ L^{-1}$)	Source	Comments
10 μM	Si/BDD	96	50 mM Na_2SO_4	180 mA	0.366	0.4	Our study	Optimum BA degradation
10 μM	Nb/BDD	64	10 mM Na_2SO_4	90 mA	0.262	0.3	Our study	Taguchi 1
10 μM	Nb/BDD	64	50 mM Na_2SO_4	180 mA	0.368	0.4	Our study	Taguchi 2
10 μM	Si/BDD	96	50 mM Na_2SO_4	90 mA	0.299	0.3	Our study	Taguchi 3
10 μM	Si/BDD	96	10 mM Na_2SO_4	180 mA	0.337	0.3	Our study	Taguchi 4
10 μM	Nb/BDD-Ag	64	50 mM Na_2SO_4	90 mA	0.146	0.2	Our study	Taguchi 5
10 μM	Nb/BDD-Ag	64	10 mM Na_2SO_4	180 mA	0.205	0.2	Our study	Taguchi 6
150 mg L^{-1}	BDD	70	50 mM Na_2SO_4	18 A	-	39.0	[1]	Flow cell, initial pH 3.8
150 mg L^{-1}	BDD	70	50 mM Na_2SO_4	18 A	-	13.0	[1]	Flow cell, initial pH 10
10 mM	BDD	10	400 mM Na_2SO_4, 100 mM NaH_2PO_4, 100 mM Na_2HPO_4	2.25 V (2.5 mA cm^{-2})	-	341.9	[2]	Flow cell, initial pH 6
50 mg L^{-1}	PbO_2	-	100 mM Na_2SO_4	150 mA cm^{-2}	0.0259	1.3	[3]	Different electrode

References

[1] Velegraki T, Balayiannis G, Diamadopoulos E, Katsaounis A, Mantzavinos D. Electrochemical oxidation of benzoic acid in water over boron-doped diamond electrodes: Statistical analysis of key operating parameters, kinetic modeling, reaction by-products and ecotoxicity. Chem Eng J. 2010;160(2):538-48.

[2] Arts A, van den Berg KP, de Groot MT, van der Schaaf J. Electrochemical oxidation of benzoic acid and its aromatic intermediates on boron doped diamond electrodes. Curr Res Green Sustain Chem. 2021;4:100217.

[3] He Z, Hayat MD, Huang S, Wang X, Cao P. PbO_2 electrodes prepared by pulse reverse electrodeposition and their application in benzoic acid degradation. J Electroanal Chem. 2018;812:74-81.
